# Supplementary material for: Extracellular Proteome Analysis Shows the Abundance of Histidine Kinase Sensor Protein, DNA Helicase, Putative Lipoprotein Containing Peptidase M75 Domain and Peptidase C39 Domain Protein in Leptospira interrogans Grown in EMJH Medium
Source: Pathogens. 2021 Jul 6;10(7):852. doi: 10.3390/pathogens10070852 (PMC8308593; doi:10.3390/pathogens10070852)
Supplement: Supplementary file 1 [file pathogens-10-00852-s001.zip › pathogens-1260330-supplementary.pdf]

Extracellular proteome analysis shows the abundance of Histidine kinase sensor protein (LIC_11528), DNA helicase (LIC_11624), Putative lipoprotein (LIC_10713) and Peptidase C39 domain protein (LIC_10511) in Leptospira interrogans grown in EMJH medium.

(Abhijit. Sarma, Dhandapani. Gunasekaran, D A B. Rex, Thoduvayil. Sikha, K Mangalaparthi. Kiran, M Pinto. Sneha, Prasad T S. Keshava, Madanan. Madathiparambil)

Correspondence: Email: madanan.mg@icmr.gov.in

Supplementary Table S1

List of proteins identified in LC-MS/MS: Calculation of abundance: to determine extracellular proteins, Localization: using PSORTdb 3.0 & CELLO V.2.5 and BUSCA and Prediction of Pathogenicity: using MP3.

Legends

Abundance: W = Wash and S = Culture Supernatant. Virulence Prediction MP3: P = Pathogenic, NP = Nonpathogenic.

Localization: PSORTdb 3.0 & CELLO V.2.5: CY = Cytoplasmic, EC = Extracellular, IM = Inner membrane/Cytoplasmic Membrane, OM = Outer Membrane, PP = Periplasmic, UN = Unknown. BUSCA GO-IDs: GO:0005737 = Cytoplasmic = CY, GO:0019867 = Outer membrane = OM, GO:0005886 = Plasma membrane PM, GO:0005615 = Extracellular = EC

Pathogenicity (MP3): P = Pathogenic, NP = Nonpathogenic

Sl. No.	Accession (RefSeq)	Gene names (ordered locus)	Description	C= (Total value of Triton X-114 fractions)	Wash (W) (Surface)	Supernatant (S) (Secretory)	Abundance of Extracellular Proteins (EC) = (W+S)/C	PSORTdb 3.0	CELLO v.2.5	BUSCA_GO-ID	Pathogenicity (MP3)
1	WP_000424814.1	_	ComF family protein	NF	8.4	NF	Ex. W	CY	CY	GO:0005737	NP
2	WP_000486779.1	LIC_10697	hypothetical protein	NF	2.2	NF	Ex. W	UN	CY	GO:0005737	P

3	WP_001973446.1	_	hypothetical protein	NF	0.7	NF	Ex. W	UN	CY	GO:0005737	P
4	WP_000700529.1	_	hypothetical protein	NF	0.5	NF	Ex. W	UN	PP	GO:0005737	P
5	WP_000865345.1	LIC_13053	fatty acid desaturase	NF	0.3	NF	Ex. W	IM	IM	GO:0005886	NP
6	WP_000286526.1	_	hypothetical protein	NF	0.1	NF	Ex. W	UN	OM	GO:0005886	P
7	WP_001021444.1	_	DUF1563 domain-containing protein	NF	NF	18.2	Ex. S	UN	CY	GO:0005737	NP
8	WP_025176658.1	_	hypothetical protein	NF	NF	17.3	Ex. S	UN	CY	GO:0019867	NP
9	WP_000738678.1	LIC_12988	lipase	NF	NF	14.3	Ex. S	EC	EC	GO:0005886	NP
10	WP_000371179.1	LIC_13177	hypothetical protein	NF	NF	5.9	Ex. S	CY	CY	GO:0005737	P
11	WP_001088866.1	LIC_12191	NUDIX hydrolase	NF	NF	2.3	Ex. S	UN	CY	GO:0005737	NP
12	WP_000899352.1	_	sphingomyelin phosphodiesterase	NF	NF	1.7	Ex. S	EC	EC	GO:0005737	P
13	WP_000812418.1	LIC_10645	hypothetical protein	NF	NF	1.4	Ex. S	UN	EC	GO:0005886	P
14	WP_000844347.1	LIC_10346	SGNH/GDSL hydrolase family protein	NF	NF	1.2	Ex. S	UN	OM	GO:0005737	NP
15	WP_000416054.1	LIC_11345	TonB-dependent siderophore receptor	NF	NF	0.0	Ex. S	OM	OM	GO:0005886	P
16	WP_001193644.1	_	hypothetical protein	NF	2.1	4.7	Ex. W&S	IM	IM	GO:0005886	P
17	WP_000336237.1	LIC_12715	DUF1561 domain-containing protein	NF	0.7	3.2	Ex. W&S	UN	EC	GO:0005886	P
18	WP_000533970.1	LIC_12986	DUF1561 domain-containing protein	NF	0.5	3.9	Ex. W&S	EC	OM	GO:0005886	P
19	WP_001211842.1	LIC_11088	di-heme enzyme	NF	0.4	4.8	Ex. W&S	PP	EC	GO:0005737	P

20	WP_000433639.1	LIC_11528	PAS domain S-box protein	0.5	941.1	7.0	1771.4	IM	CY	GO:0005737	P
21	WP_000378420.1	LIC_11624	ATPase AAA	21.5	813.4	1871.9	124.9	CY	OM	GO:0005737	NP
22	WP_001049563.1	LIC_10713	peptidase M75	23.5	11.8	1833.1	78.5	OM	OM	GO:0005886	P
23	WP_001205796.1	LIC_10511	hypothetical protein	0.1	NF	4.0	75.6	UN	PP	GO:0005737	P
24	WP_000938871.1	LIC_20255	hypothetical protein	1.7	42.3	29.6	42.5	CY	CY	GO:0005737	P
25	WP_000752033.1	LIC_10370	hypothetical protein	2.1	10.8	72.9	38.9	UN	EC	GO:0005615	P
26	WP_001071893.1	LIC_10704	hypothetical protein	2.9	NF	101.8	35.2	CY	CY	GO:0005886	NP
27	WP_000689168.1	_	SGNH/GDSL hydrolase family protein	2.0	NF	55.3	28.0	IM	EC	GO:0005615	NP
28	WP_002002492.1	LIC_11904	hypothetical protein	3.3	NF	86.1	26.3	IM	OM	GO:0005886	P
29	WP_001010056.1	_	hypothetical protein	3.6	NF	94.0	26.2	CY	OM	GO:0005886	NP
30	WP_000141324.1	LIC_11265	MULTISPECIES: DUF1858 domain-containing protein	5.4	NF	139.8	25.9	UN	CY	GO:0005737	NP
31	WP_000620240.1	LIC_10371	hypothetical protein	12.1	2.4	246.8	20.7	UN	OM	GO:0005615	P
32	WP_080011816.1	_	sphingomyelin phosphodiesterase	0.4	NF	7.0	19.4	EC	OM	GO:0005737	P
33	WP_000806224.1	LIC_13164	2-amino-4-hydroxy-6-hydroxymethyldihydropteridine diphosphokinase	11.1	1.0	206.8	18.6	CY	CY	GO:0005737	NP
34	WP_000768408.1	LIC_12891	hypothetical protein	2.2	1.6	35.9	16.7	IM	EC	GO:0005886	P
35	WP_000680185.1	LIC_10365	DUF1565 domain-containing protein	0.6	1.3	7.6	14.7	UN	EC	GO:0005615	P
36	WP_000620247.1	LIC_10373	hypothetical protein	4.6	2.5	65.8	14.7	UN	EC	GO:0005886	P

37	WP_000564696.1	LIC_10183	hypothetical protein	0.8	11.0	NF	14.4	CY	CY	GO:0005737	NP
38	WP_000999999.1	LIC_11240	ATP synthase subunit delta	205.7	0.7	2731.9	13.3	CY	CY	GO:0005737	NP
39	WP_000696002.1	LIC_13248	DUF1554 domain-containing protein	0.7	NF	8.6	12.6	UN	EC	GO:0005615	P
40	WP_000092385.1	LIC_12791	DUF1561 domain-containing protein	0.6	NF	6.7	11.7	UN	OM	GO:0005886	P
41	WP_000019139.1	LIC_12413	DUF115 domain-containing protein	3.4	NF	38.3	11.2	UN	OM	GO:0005737	NP
42	WP_000603280.1	LIC_20247	lytic transglycosylase domain-containing protein	0.7	NF	6.9	10.3	PP	CY	GO:0005737	NP
43	WP_000734588.1	_	hypothetical protein	11.2	NF	92.3	8.2	EC	OM	GO:0005886	P
44	WP_000440098.1	_	DUF2203 domain-containing protein	3.1	20.5	NF	6.6	CY	CY	GO:0005737	P
45	WP_000141830.1	LIC_12540	MULTISPECIES: preprotein translocase subunit YajC	1067.4	1534.6	4361.7	5.5	IM	CY	GO:0005886	NP
46	WP_001001307.1	_	hypothetical protein	0.5	2.4	NF	5.2	EC	EC	GO:0005737	P
47	WP_001019268.1	LIC_12755	MULTISPECIES: hypothetical protein	94.8	21.7	289.9	3.3	CY	CY	GO:0005737	NP
48	WP_001005911.1	LIC_10552	hybrid sensor histidine kinase/response regulator	0.8	NF	2.7	3.2	IM	CY	GO:0005737	NP
49	WP_000587664.1	_	VOC family protein	198.7	35.6	428.8	2.3	CY	CY	GO:0005737	P
50	WP_000658301.1	_	DNA starvation/stationary phase protection protein	33.3	54.1	22.1	2.3	CY	CY	GO:0005886	P
51	WP_000278813.1	_	DUF192 domain-containing protein	2.0	2.9	1.2	2.1	UN	CY	GO:0005737	NP
52	WP_001221057.1	LIC_10711	hypothetical protein	5.1	8.7	NF	1.7	OM	OM	GO:0005886	P
53	WP_001007329.1	LIC_12437	hypothetical protein	94.4	8.0	151.1	1.7	CY	CY	GO:0005737	NP

54	WP_000464920.1	_	stage II sporulation protein E	2.6	NF	4.1	1.6	IM	IM	GO:0005886	NP
55	WP_000478308.1	_	sphingomyelin phosphodiesterase	1.8	0.7	2.1	1.6	EC	OM	GO:0005737	P
56	WP_000240010.1	LIC_11555	30S ribosomal protein S16	27.9	NF	42.3	1.5	CY	PP	GO:0005737	NP
57	WP_000015950.1	LIC_12932	DUF1292 domain-containing protein	12.5	NF	17.2	1.4	CY	CY	GO:0005737	P
58	WP_000927928.1	LIC_11775	phosphoserine phosphatase SerB	10.1	NF	13.6	1.3	CY	CY	GO:0005737	NP
59	WP_000124499.1	LIC_12869	30S ribosomal protein S19	93.9	NF	116.0	1.2	CY	PP	GO:0005737	NP
60	WP_000280002.1	LIC_10695	DUF1561 domain-containing protein	0.2	NF	0.2	1.2	EC	EC	GO:0005615	P
61	WP_000653315.1	LIC_10902	hypothetical protein	0.2	0.3	NF	1.2	UN	OM	GO:0005886	NP
62	WP_000657852.1	_	glutathione peroxidase	56.8	54.9	9.9	1.1	PP	PP	GO:0005886	P
63	WP_000918607.1	LIC_12874	MULTISPECIES: 30S ribosomal protein S10	187.1	NF	205.3	1.1	CY	CY	GO:0005737	NP
64	WP_000600072.1	_	hypothetical protein	0.5	NF	0.5	1.1	UN	OM	GO:0005886	P
65	WP_000753030.1	LIC_20065	MULTISPECIES: acyl carrier protein	11098.3	3248.0	8477.5	1.1	CY	CY	GO:0005737	NP
66	WP_000279228.1	LIC_12706	transcription termination/antitermination protein NusA	158.1	70.7	93.9	1.0	CY	CY	GO:0005737	NP
67	WP_000653730.1	_	hypothetical protein	7.0	6.8	NF	1.0	UN	CY	GO:0005737	NP
68	WP_001983274.1	_	DUF1554 domain-containing protein	2.9	2.8	NF	1.0	UN	EC	GO:0005615	P
69	WP_000692278.1	LIC_10827	hypothetical protein	2.6	1.0	1.5	1.0	UN	OM	GO:0005615	P
70	WP_000426654.1	LIC_12453	segregation and condensation protein A	11.7	0.0	10.9	0.9	CY	CY	GO:0005737	NP
71	WP_001009586.1	LIC_11388	transporter	16.0	14.8	NF	0.9	UN	EC	GO:0005737	P

72	WP_001097652.1	LIC_11221	histidine kinase	4.3	NF	3.9	0.9	CY	CY	GO:0005737	P
73	WP_001192276.1	LIC_11075	NAD(+) synthase	6.7	0.6	5.5	0.9	CY	OM	GO:0005886	NP
74	WP_000791241.1	LIC_20176	DUF1566 domain-containing protein	9.5	4.5	4.1	0.9	UN	EC	GO:0005886	P
75	WP_000836548.1	LIC_13152	D-alanyl-D-alanine carboxypeptidase	51.5	46.5	NF	0.9	CY	OM	GO:0005615	P
76	WP_000570772.1	_	SET domain-containing protein-lysine N-methyltransferase	123.6	NF	109.5	0.9	CY	CY	GO:0005737	NP
77	WP_000695952.1	LIC_13071	YbhB/YbcL family Raf kinase inhibitor-like protein	339.1	298.2	0.9	0.9	PP	PP	GO:0005615	P
78	WP_000683633.1	LIC_11048	WGR domain-containing protein	22.9	0.5	19.6	0.9	CY	CY	GO:0005737	NP
79	WP_000443314.1	_	MULTISPECIES: OmpA family protein	56.4	34.5	13.4	0.8	UN	CY	GO:0005737	NP
80	WP_001253849.1	_	lipoprotein LipL36	8134.9	4384.3	2385.7	0.8	UN	EC	GO:0005615	P
81	WP_002144337.1	_	hypothetical protein	4.8	4.0	NF	0.8	EC	CY	GO:0005737	P
82	WP_000932549.1	LIC_11035	DUF4087 domain-containing protein	167.2	101.0	35.6	0.8	UN	PP	GO:0005615	P
83	WP_000913838.1	LIC_10705	hypothetical protein	27.1	3.9	18.0	0.8	UN	EC	GO:0005615	P
84	WP_000768559.1	LIC_11903	hypothetical protein	2.9	NF	2.3	0.8	PP	PP	GO:0005886	P
85	WP_000515894.1	_	MULTISPECIES: S-adenosylmethionine decarboxylase proenzyme	111.0	6.0	81.8	0.8	UN	CY	GO:0005737	NP
86	WP_000275939.1	_	sphingomyelin phosphodiesterase	0.0	0.0	NF	0.8	EC	OM	GO:0005737	P
87	WP_002139006.1	_	DUF1566 domain-containing protein	15.3	11.8	0.2	0.8	UN	EC	GO:0005737	P
88	WP_001020567.1	LIC_10046	NAD(P)(+) transhydrogenase (Re/Si-specific) subunit alpha	1059.7	757.3	61.3	0.8	IM	PP	GO:0005737	NP

89	WP_001245964.1	LIC_12963	DUF1561 domain-containing protein	5.6	NF	4.2	0.7	UN	EC	GO:0005615	P
90	WP_002145430.1	_	glucose sorbosone dehydrogenase	16.4	12.0	NF	0.7	CY	CY	GO:0005737	NP
91	WP_001081802.1	LIC_12680	DUF1566 domain-containing protein	0.8	NF	0.6	0.7	UN	OM	GO:0005615	P
92	WP_000760764.1	LIC_12253	MULTISPECIES: DUF1566 domain-containing protein	23.5	15.9	0.9	0.7	UN	OM	GO:0005737	P
93	WP_000657656.1	LIC_12609	hypothetical protein	2.3	NF	1.7	0.7	CY	CY	GO:0005737	NP
94	WP_000577467.1	LIC_12634	hypothetical protein	1.2	NF	0.8	0.7	CY	CY	GO:0005737	NP
95	WP_000673420.1	LIC_11424	FKBP-type peptidyl-prolyl cis-trans isomerase	367.7	260.1	0.5	0.7	PP	CY	GO:0005615	NP
96	WP_000275600.1	LIC_12236	MULTISPECIES: CBS domain-containing protein	15.2	NF	10.5	0.7	CY	CY	GO:0005737	NP
97	WP_000478876.1	LIC_12262	hypothetical protein	2.4	NF	1.6	0.7	UN	OM	GO:0005737	NP
98	WP_001975119.1	_	Cys-rich protein	504.9	348.3	NF	0.7	UN	PP	GO:0005615	P
99	WP_000713555.1	_	hypothetical protein	17.0	1.9	9.7	0.7	IM	EC	GO:0005615	P
100	WP_001263864.1	LIC_12110	50S ribosomal protein L9	171.0	NF	116.7	0.7	CY	CY	GO:0005737	NP
101	WP_000842617.1	LIC_12529	sulfate ABC transporter substrate-binding protein	134.8	91.4	NF	0.7	PP	CY	GO:0005886	NP
102	WP_000615243.1	LIC_10561	YHYH protein	26.9	NF	18.1	0.7	EC	EC	GO:0005615	P
103	WP_001115288.1	LIC_11951	ATP-dependent Clp protease proteolytic subunit	92.3	8.5	53.3	0.7	CY	CY	GO:0005737	NP
104	WP_000695170.1	LIC_10712	thiol oxidoreductase	6.7	NF	4.4	0.7	UN	OM	GO:0005886	P

105	WP_001040194.1	LIC_12851	MULTISPECIES: translation initiation factor IF-1	56.9	37.7	NF	0.7	CY	CY	GO:0005737	NP
106	WP_001087680.1	LIC_10793	hypothetical protein	2106.0	1324.5	1.2	0.6	CY	CY	GO:0005737	NP
107	WP_000682355.1	LIC_12561	hypothetical protein	39.8	25.1	NF	0.6	UN	PP	GO:0005615	P
108	WP_000103483.1	LIC_10175	hypothetical protein	89.5	NF	55.9	0.6	CY	CY	GO:0005737	NP
109	WP_000162390.1	LIC_11082	hypothetical protein	0.6	NF	0.4	0.6	CY	OM	GO:0005737	NP
110	WP_000381195.1	LIC_12724	hypothetical protein	2.6	NF	1.5	0.6	CY	CY	GO:0005737	NP
111	WP_000378482.1	LIC_12490	DNA-directed RNA polymerase sigma-70 factor	53.0	NF	30.5	0.6	CY	CY	GO:0005737	NP
112	WP_000029425.1	LIC_20142	adenine phosphoribosyltransferase	24.8	NF	14.1	0.6	CY	CY	GO:0005737	NP
113	WP_000810437.1	_	DUF3347 domain-containing protein	88.5	49.9	NF	0.6	UN	PP	GO:0005886	NP
114	WP_001250026.1	LIC_12377	metallophosphatase	245.3	130.9	3.6	0.5	IM	PP	GO:0005615	P
115	WP_001087205.1	LIC_12341	hypothetical protein	124.9	59.3	7.6	0.5	UN	CY	GO:0005886	P
116	WP_001054325.1	LIC_11780	8-amino-7-oxononanoate synthase	19.9	8.2	2.4	0.5	CY	OM	GO:0005737	NP
117	WP_001054050.1	LIC_10225	MULTISPECIES: sigma-70 family RNA polymerase sigma factor	157.3	NF	81.9	0.5	CY	CY	GO:0005737	NP
118	WP_000660863.1	LIC_12743	NADH dehydrogenase	108.0	7.0	46.9	0.5	CY	CY	GO:0005737	NP
119	WP_000424879.1	LIC_11652	MULTISPECIES: transaldolase	955.8	10.5	460.8	0.5	CY	CY	GO:0005737	NP
120	WP_000472806.1	_	hypothetical protein	654.2	285.5	30.2	0.5	UN	OM	GO:0005615	P
121	WP_000120541.1	_	hypothetical protein	8.6	1.9	2.2	0.5	EC	OM	GO:0005737	P

122	WP_000270004.1	_	hypothetical protein	16.1	7.0	0.7	0.5	UN	CY	GO:0005615	NP
123	WP_000623889.1	_	hypothetical protein	49.6	23.7	NF	0.5	CY	CY	GO:0005615	NP
124	WP_000868876.1	LIC_10916	geranylgeranyl pyrophosphate synthase	5.9	2.8	NF	0.5	CY	CY	GO:0005737	NP
125	WP_000649940.1	LIC_10769	insulinase family protein	78.6	33.1	3.2	0.5	CY	OM	GO:0005886	NP
126	WP_000721126.1	_	hypothetical protein	157.7	49.3	22.8	0.5	UN	PP	GO:0005615	NP
127	WP_001162965.1	LIC_10176	hypothetical protein	90.2	2.4	38.0	0.4	UN	CY	GO:0005737	NP
128	WP_000692782.1	_	hypothetical protein	47.9	12.4	8.2	0.4	UN	OM	GO:0005886	P
129	WP_001049247.1	_	hypothetical protein	503.8	199.9	10.8	0.4	UN	CY	GO:0005737	P
130	WP_020423544.1	_	hypothetical protein	2.8	NF	1.2	0.4	OM	OM	GO:0005737	P
131	WP_001262509.1	LIC_12112	single-stranded DNA-binding protein	256.8	12.0	93.0	0.4	CY	EC	GO:0005737	NP
132	WP_002120897.1	LIC_11958	hypothetical protein	118.8	46.5	NF	0.4	CY	CY	GO:0005886	NP
133	WP_000680192.1	LIC_13131	YceI family protein	12.7	4.9	NF	0.4	UN	CY	GO:0005615	NP
134	WP_000680555.1	LIC_11410	acetolactate synthase small subunit	125.9	43.7	4.8	0.4	CY	CY	GO:0005737	NP
135	WP_002082849.1	_	SH3 domain-containing protein	81.5	30.9	NF	0.4	CY	CY	GO:0005886	P
136	WP_000348938.1	LIC_12505	REC domain-containing phosphodiesterase	34.1	NF	12.9	0.4	IM	CY	GO:0005737	NP
137	WP_000682546.1	LIC_13253	hypothetical protein	92.1	34.6	NF	0.4	UN	PP	GO:0005615	P
138	WP_001158185.1	LIC_11175	S-methyl-5-thioribose-1-phosphate isomerase	8.7	NF	3.3	0.4	CY	CY	GO:0005737	NP
139	WP_000055098.1	LIC_13396	monooxygenase	0.3	0.1	NF	0.4	CY	CY	GO:0005737	NP

140	WP_000005312.1	LIC_12400	isoleucine--tRNA ligase	380.9	128.0	12.4	0.4	CY	CY	GO:0005737	NP
141	WP_001221434.1	LIC_20197	cysteine protease	140.1	16.1	33.9	0.4	OM	OM	GO:0005737	P
142	WP_000720585.1	_	hypothetical protein	7.4	2.6	NF	0.4	UN	CY	GO:0005615	P
143	WP_000821257.1	LIC_10760	YceI family protein	375.9	104.8	24.8	0.3	UN	EC	GO:0005615	P
144	WP_082273108.1	_	TIGR04388 family protein	0.3	NF	0.1	0.3	UN	EC	GO:0005886	NP
145	WP_000798837.1	LIC_12812	PDZ domain-containing protein	211.1	38.6	32.8	0.3	PP	PP	GO:0005886	NP
146	WP_000449766.1	LIC_10582	anthranilate synthase component I	132.0	2.5	41.8	0.3	CY	CY	GO:0005737	NP
147	WP_000283005.1	_	DUF1561 domain-containing protein	7.1	NF	2.4	0.3	EC	EC	GO:0005737	P
148	WP_001011312.1	LIC_10828	hypothetical protein	10.9	3.6	NF	0.3	EC	CY	GO:0005737	P
149	WP_001274793.1	LIC_12245	MULTISPECIES: RNA-binding protein Hfq	1099.1	274.3	84.0	0.3	CY	CY	GO:0005737	NP
150	WP_000532666.1	LIC_12286	hypothetical protein	1.1	0.4	NF	0.3	UN	OM	GO:0005737	P
151	WP_000270913.1	LIC_12776	MULTISPECIES: 50S ribosomal protein L21	732.6	NF	236.5	0.3	CY	PP	GO:0005737	NP
152	WP_000974282.1	LIC_12621	MULTISPECIES: polymer-forming cytoskeletal protein	4292.6	1317.3	62.5	0.3	CY	CY	GO:0005737	NP
153	WP_000674082.1	LIC_10512	hypothetical protein	244.6	78.6	NF	0.3	CY	CY	GO:0005615	P
154	WP_000701613.1	LIC_10995	alpha/beta hydrolase	17.7	5.6	NF	0.3	UN	OM	GO:0005886	NP
155	WP_000834071.1	LIC_10086	TrmA/RlmC/RlmD family RNA methyltransferase	0.9	0.3	NF	0.3	CY	OM	GO:0005737	NP
156	WP_000143898.1	LIC_13381	carotenoid 1,2-hydratase	1.7	0.5	NF	0.3	UN	OM	GO:0005886	P
157	WP_001081432.1	LIC_10241	hypothetical protein	33.9	1.6	8.9	0.3	CY	CY	GO:0005737	NP

158	WP_000870126.1	LIC_10958	NAD(P)-dependent alcohol dehydrogenase	377.6	116.4	0.4	0.3	CY	CY	GO:0005737	NP
159	WP_000281020.1	LIC_12412	chromosome segregation ATPase	20.1	NF	6.2	0.3	CY	CY	GO:0005737	NP
160	WP_000007538.1	LIC_12146	polysaccharide deacetylase	8.2	2.1	0.4	0.3	CY	CY	GO:0005737	NP
161	WP_001202539.1	LIC_11915	thiamine phosphate synthase	4.7	0.7	0.6	0.3	CY	CY	GO:0005737	NP
162	WP_000443344.1	LIC_10095	hypothetical protein	340.7	100.1	NF	0.3	UN	CY	GO:0005886	P
163	WP_000444619.1	LIC_12326	DUF1987 domain-containing protein	245.1	63.8	7.9	0.3	UN	CY	GO:0005737	P
164	WP_000615917.1	LIC_12863	MULTISPECIES: 50S ribosomal protein L14	168.2	NF	49.0	0.3	CY	CY	GO:0005737	NP
165	WP_000940638.1	LIC_11720	hydrolase	136.8	16.0	23.7	0.3	UN	OM	GO:0005886	NP
166	WP_000868567.1	LIC_12816	hypothetical protein	56.3	13.0	3.4	0.3	UN	CY	GO:0005886	NP
167	WP_001974248.1	_	malate dehydrogenase	38.4	0.3	10.8	0.3	CY	CY	GO:0005737	NP
168	WP_000039575.1	LIC_11112	serine protease	23.3	4.3	2.4	0.3	UN	OM	GO:0005615	NP
169	WP_000367409.1	LIC_10268	MULTISPECIES: Crp/Fnr family transcriptional regulator	86.2	NF	24.8	0.3	CY	CY	GO:0005737	NP
170	WP_000581246.1	LIC_13043	hypothetical protein	6.8	1.9	NF	0.3	UN	CY	GO:0005737	P
171	WP_001040571.1	LIC_12875	elongation factor Tu	8211.9	628.2	1700.2	0.3	CY	CY	GO:0005737	NP
172	WP_001043223.1	_	DUF2147 domain-containing protein	913.1	258.7	NF	0.3	UN	PP	GO:0005615	NP
173	WP_001070403.1	LIC_13279	thiolase family protein	56.8	15.9	0.1	0.3	CY	OM	GO:0005737	NP
174	WP_001160150.1	LIC_12335	serine hydroxymethyltransferase	205.4	13.8	43.3	0.3	CY	PP	GO:0005737	NP
175	WP_001070615.1	_	hypothetical protein	19.9	1.5	3.9	0.3	UN	CY	GO:0005886	P

176	WP_080011824.1	_	peptidase M28	1.3	0.3	NF	0.3	EC	CY	GO:0005737	NP
177	WP_000502132.1	_	hypothetical protein	103.9	25.6	2.5	0.3	UN	PP	GO:0005737	NP
178	WP_000409822.1	LIC_12402	phosphoribosylformylglycinamidine synthase subunit PurL	140.5	0.6	36.8	0.3	CY	CY	GO:0005737	NP
179	WP_001283178.1	_	MULTISPECIES: thioredoxin-dependent thiol peroxidase	231.8	61.2	NF	0.3	CY	PP	GO:0005737	P
180	WP_000470547.1	_	hypothetical protein	2.8	0.7	NF	0.3	CY	CY	GO:0005886	NP
181	WP_000333575.1	LIC_10768	insulinase family protein	13.7	2.1	1.4	0.3	CY	CY	GO:0005886	NP
182	WP_000146552.1	LIC_11336	MULTISPECIES: molecular chaperone GroES	4397.5	985.6	150.5	0.3	CY	CY	GO:0005737	NP
183	WP_000619331.1	LIC_13494	hypothetical protein	46.8	11.8	NF	0.3	UN	EC	GO:0005886	P
184	WP_000005292.1	LIC_12025	asparagine--tRNA ligase	294.6	6.7	67.5	0.3	CY	CY	GO:0005737	NP
185	WP_000790723.1	LIC_12303	MULTISPECIES: S41 family peptidase	159.7	40.1	NF	0.3	IM	OM	GO:0005737	NP
186	WP_000116457.1	_	PaaI family thioesterase	11.2	NF	2.8	0.3	IM	PP	GO:0005737	NP
187	WP_000949351.1	LIC_12263	hypothetical protein	343.8	54.7	31.1	0.2	CY	CY	GO:0005886	P
188	WP_000620907.1	LIC_11334	SIMPL domain-containing protein	298.5	69.8	4.5	0.2	UN	OM	GO:0005615	P
189	WP_001968918.1	_	polysaccharide deacetylase family protein	46.7	9.5	2.1	0.2	UN	PP	GO:0005615	P
190	WP_001075122.1	LIC_10655	hypothetical protein	134.4	33.0	NF	0.2	UN	PP	GO:0005886	P
191	WP_000742409.1	LIC_13355	LIC_13355 family lipoprotein	44.6	10.9	NF	0.2	UN	EC	GO:0005615	P
192	WP_000648186.1	LIC_11209	MULTISPECIES: PaaI family thioesterase	754.0	55.4	129.0	0.2	CY	CY	GO:0005737	NP
193	WP_001032649.1	LIC_11957	response regulator	41.0	NF	9.9	0.2	CY	CY	GO:0005737	NP

194	WP_000635440.1	LIC_10114	1-(5-phosphoribosyl)-5-((5-phosphoribosylamino)methylideneamino)imidazole-4-carboxamide isomerase	9.1	0.9	1.3	0.2	CY	CY	GO:0005737	NP
195	WP_000927327.1	_	S41 family peptidase	46.9	0.3	10.9	0.2	IM	CY	GO:0005615	NP
196	WP_001188705.1	LIC_13293	peptidase	36.9	4.4	4.3	0.2	UN	OM	GO:0005615	NP
197	WP_000842388.1	_	peptidylprolyl isomerase	426.4	8.5	90.7	0.2	CY	CY	GO:0005737	NP
198	WP_001136837.1	LIC_12461	50S ribosomal protein L20	297.8	NF	69.2	0.2	CY	CY	GO:0005737	NP
199	WP_000531352.1	LIC_12024	hypothetical protein	36.5	NF	8.5	0.2	CY	CY	GO:0005737	NP
200	WP_000201605.1	LIC_11835	MULTISPECIES: ferredoxin	441.6	101.4	0.7	0.2	UN	CY	GO:0005737	NP
201	WP_001974506.1	LIC_11197	MULTISPECIES: pyridoxamine 5'-phosphate oxidase family protein	759.9	11.1	161.4	0.2	CY	CY	GO:0005737	NP
202	WP_000802156.1	LIC_11821	3-isopropylmalate dehydratase small subunit	384.5	44.5	42.6	0.2	CY	CY	GO:0005737	NP
203	WP_001002663.1	_	transketolase	103.4	1.1	22.3	0.2	CY	CY	GO:0005737	NP
204	WP_000570763.1	LIC_11964	MULTISPECIES: hypothetical protein	55.3	NF	12.5	0.2	UN	CY	GO:0019867	NP
205	WP_000369357.1	_	7-carboxy-7-deazaguanine synthase	12.4	NF	2.8	0.2	CY	CY	GO:0005737	NP
206	WP_000736494.1	LIC_11352	MULTISPECIES: outer membrane surface lipoprotein LipL32	6388.2	895.4	542.7	0.2	UN	PP	GO:0005615	P
207	WP_001206943.1	LIC_13300	MULTISPECIES: 3-hydroxyacyl-CoA dehydrogenase	432.6	19.3	77.7	0.2	CY	CY	GO:0005737	NP
208	WP_000750243.1	LIC_10579	response regulator	15.4	NF	3.4	0.2	UN	CY	GO:0005737	P

209	WP_033108072.1	_	septal ring lytic transglycosylase RlpA family protein	104.5	23.2	NF	0.2	UN	OM	GO:0005615	NP
210	WP_000125527.1	LIC_20175	molecular chaperone DnaK	350.1	67.2	10.6	0.2	CY	CY	GO:0005737	NP
211	WP_000074962.1	LIC_12738	MarR family transcriptional regulator	13.9	NF	3.0	0.2	UN	CY	GO:0005737	NP
212	WP_000545443.1	LIC_12730	hypothetical protein	92.9	11.7	8.4	0.2	UN	OM	GO:0005615	P
213	WP_000795862.1	LIC_11027	hypothetical protein	11.6	2.5	NF	0.2	UN	PP	GO:0005615	P
214	WP_000845498.1	LIC_20080	acyl-CoA thioesterase	19.9	4.3	NF	0.2	UN	CY	GO:0005737	NP
215	WP_000371581.1	LIC_11263	hypothetical protein	7.1	NF	1.5	0.2	CY	CY	GO:0005737	NP
216	WP_001005189.1	LIC_11595	hypothetical protein	28.1	0.5	5.5	0.2	CY	CY	GO:0005737	NP
217	WP_001109419.1	LIC_12759	MULTISPECIES: membrane protein	52.8	11.3	NF	0.2	EC	OM	GO:0005737	P
218	WP_001970524.1	LIC_11978	MULTISPECIES: thioredoxin	435.3	93.0	0.1	0.2	CY	CY	GO:0005737	NP
219	WP_001291837.1	LIC_13328	isocitrate dehydrogenase	126.8	9.1	17.9	0.2	CY	CY	GO:0005737	NP
220	WP_000364507.1	LIC_11539	D-glycero-beta-D-manno-heptose 1-phosphate adenylyltransferase	13.8	NF	2.9	0.2	CY	CY	GO:0005737	NP
221	WP_000680361.1	LIC_12481	N-acetyltransferase	169.0	7.7	28.0	0.2	CY	CY	GO:0005737	P
222	WP_000443450.1	LIC_12773	GTPase ObgE	14.2	0.5	2.5	0.2	CY	CY	GO:0005737	NP
223	WP_001011107.1	LIC_11098	membrane protein	26.2	5.5	NF	0.2	EC	EC	GO:0005737	P
224	WP_000778114.1	LIC_13353	NAD(P)/FAD-dependent oxidoreductase	79.3	15.6	1.1	0.2	UN	OM	GO:0005737	NP
225	WP_000054457.1	LIC_12878	thioredoxin family protein	249.2	20.6	31.5	0.2	CY	CY	GO:0005737	P
226	WP_001074459.1	LIC_20250	OmpA family protein	90.6	18.8	NF	0.2	OM	CY	GO:0005737	NP

227	WP_000692533.1	LIC_13147	phosphoesterase	20.5	0.3	4.0	0.2	CY	CY	GO:0005737	NP
228	WP_000462055.1	LIC_13478	ABC transporter substrate-binding protein	69.4	12.9	1.4	0.2	PP	OM	GO:0005615	P
229	WP_000957839.1	LIC_11442	hypothetical protein	90.8	17.9	0.7	0.2	UN	PP	GO:0005737	NP
230	WP_000944569.1	LIC_20144	PDZ domain-containing protein	238.5	37.3	11.5	0.2	CY	OM	GO:0005615	NP
231	WP_001167022.1	LIC_11488	MULTISPECIES: chemotaxis protein CheX	39.6	NF	8.1	0.2	UN	CY	GO:0005737	NP
232	WP_001164879.1	LIC_11431	MULTISPECIES: cyclic nucleotide-binding domain-containing protein	410.5	83.7	NF	0.2	CY	CY	GO:0005737	NP
233	WP_000809922.1	LIC_13070	hypothetical protein	108.4	6.1	15.9	0.2	UN	OM	GO:0005615	P
234	WP_000704154.1	LIC_10846	MULTISPECIES: holo-ACP synthase	21.4	NF	4.3	0.2	CY	CY	GO:0005737	NP
235	WP_000479265.1	LIC_10581	type 1 glutamine amidotransferase	42.0	NF	8.4	0.2	CY	CY	GO:0005737	NP
236	WP_000716861.1	LIC_11722	hypothetical protein	6.0	1.2	NF	0.2	UN	CY	GO:0005615	P
237	WP_000002920.1	LIC_13075	DUF1566 domain-containing protein	4.9	1.0	NF	0.2	UN	EC	GO:0005886	P
238	WP_001096786.1	LIC_11394	MULTISPECIES: flagellar assembly protein FliH	55.2	NF	10.7	0.2	CY	CY	GO:0005737	P
239	WP_000735123.1	_	hypothetical protein	208.5	40.6	NF	0.2	UN	CY	GO:0005615	NP
240	WP_000662440.1	_	hypothetical protein	69.9	13.3	NF	0.2	OM	OM	GO:0005737	P
241	WP_000848239.1	LIC_12157	3-deoxy-manno-octulosonate cytidylyltransferase	109.4	2.3	18.4	0.2	CY	CY	GO:0005737	NP
242	WP_000852393.1	LIC_10525	nucleotide exchange factor GrpE	608.6	107.9	4.6	0.2	CY	CY	GO:0005737	NP
243	WP_000014140.1	LIC_12886	YebC/PmpR family DNA-binding transcriptional regulator	486.8	78.1	11.3	0.2	CY	CY	GO:0005737	NP

244	WP_002070403.1	_	thioesterase	19.7	3.6	NF	0.2	CY	CY	GO:0005737	NP
245	WP_000803603.1	LIC_13144	hypothetical protein	16.8	3.0	NF	0.2	CY	OM	GO:0005615	P
246	WP_000007746.1	LIC_12008	UDP-3-O-(3-hydroxymyristoyl)glucosamine N-acyltransferase	115.0	19.0	1.6	0.2	CY	CY	GO:0005737	NP
247	WP_000433083.1	LIC_12933	HNH endonuclease	32.1	5.7	NF	0.2	UN	CY	GO:0005737	NP
248	WP_000841462.1	_	gamma carbonic anhydrase family protein	62.9	2.3	8.8	0.2	CY	CY	GO:0005737	NP
249	WP_000196721.1	LIC_12090	type I glyceraldehyde-3-phosphate dehydrogenase	491.8	80.2	6.8	0.2	CY	CY	GO:0005737	NP
250	WP_000619739.1	LIC_13058	hypothetical protein	19.4	3.4	NF	0.2	CY	CY	GO:0005615	NP
251	WP_000714458.1	LIC_10520	hypothetical protein	342.0	59.8	NF	0.2	UN	EC	GO:0005615	P
252	WP_000834696.1	LIC_12075	hypothetical protein	675.9	85.6	31.9	0.2	CY	CY	GO:0005615	P
253	WP_001025523.1	LIC_10369	hypothetical protein	17.2	3.0	NF	0.2	UN	EC	GO:0005615	P
254	WP_000885546.1	LIC_10038	tetratricopeptide repeat protein	76.4	11.2	2.0	0.2	CY	CY	GO:0005615	P
255	WP_000675215.1	LIC_11111	hypothetical protein	29.9	5.1	NF	0.2	UN	OM	GO:0005737	NP
256	WP_000409256.1	LIC_10724	hypothetical protein	103.5	NF	17.7	0.2	CY	CY	GO:0005737	NP
257	WP_000359691.1	LIC_10189	hypothetical protein	4.1	NF	0.7	0.2	UN	OM	GO:0005737	P
258	WP_001293444.1	_	30S ribosomal protein S18	1503.8	26.3	226.9	0.2	CY	CY	GO:0005737	NP
259	WP_001028883.1	LIC_11985	MULTISPECIES: RNA-binding protein	1972.0	316.7	14.0	0.2	CY	CY	GO:0005737	NP
260	WP_000672221.1	LIC_10411	HEAT repeat domain-containing protein	1561.8	115.1	145.7	0.2	UN	CY	GO:0005615	P
261	WP_000331210.1	LIC_12856	MULTISPECIES: 30S ribosomal protein S5	434.1	3.1	69.0	0.2	CY	CY	GO:0005737	NP

262	WP_001968241.1	LIC_12096	hypothetical protein	23.7	3.7	0.3	0.2	CY	CY	GO:0005615	P
263	WP_001051299.1	_	hypothetical protein	822.0	54.0	79.0	0.2	CY	CY	GO:0005737	NP
264	WP_001229815.1	LIC_12821	MULTISPECIES: polymer-forming cytoskeletal protein	994.2	134.6	25.7	0.2	CY	CY	GO:0005737	P
265	WP_000601181.1	LIC_13165	cell division protein ZapE	56.7	NF	9.1	0.2	CY	CY	GO:0005737	NP
266	WP_000721620.1	LIC_11625	hypothetical protein	227.9	36.3	NF	0.2	UN	PP	GO:0005615	P
267	WP_000091691.1	LIC_13326	MULTISPECIES: nucleoside-diphosphate kinase	969.0	86.0	68.0	0.2	EC	CY	GO:0005737	NP
268	WP_000717958.1	LIC_10464	hypothetical protein	0.1	0.0	NF	0.2	UN	EC	GO:0005737	P
269	WP_000200285.1	_	MULTISPECIES: chemotaxis protein CheW	166.7	0.2	26.2	0.2	CY	CY	GO:0005737	NP
270	WP_000796624.1	LIC_11990	hypothetical protein	24.4	NF	3.8	0.2	UN	EC	GO:0005737	P
271	WP_000284396.1	LIC_10294	heme-binding protein	70.9	10.9	NF	0.2	UN	CY	GO:0005615	NP
272	WP_000844813.1	LIC_12831	SAM-dependent methyltransferase	55.5	8.5	NF	0.2	CY	CY	GO:0005737	NP
273	WP_001200127.1	_	valine--pyruvate transaminase	9.2	NF	1.4	0.2	CY	OM	GO:0005737	NP
274	WP_000669757.1	_	hypothetical protein	99.4	15.1	NF	0.2	UN	OM	GO:0005886	P
275	WP_000690686.1	LIC_12573	succinyl-CoA ligase subunit beta	1672.9	23.2	230.6	0.2	CY	CY	GO:0005737	NP
276	WP_000572090.1	LIC_11350	acyl-CoA dehydrogenase	30.9	NF	4.7	0.2	CY	CY	GO:0005737	NP
277	WP_000867010.1	_	MULTISPECIES: hypothetical protein	4486.4	216.7	462.6	0.2	UN	CY	GO:0005615	NP
278	WP_000279904.1	LIC_12574	succinate--CoA ligase subunit alpha	987.4	31.1	115.2	0.1	CY	CY	GO:0005737	NP
279	WP_000940585.1	LIC_11470	thioredoxin-disulfide reductase	220.3	32.5	NF	0.1	CY	CY	GO:0005737	NP

280	WP_001110347.1	LIC_10947	hypothetical protein	177.8	7.4	18.8	0.1	CY	CY	GO:0005737	NP
281	WP_000898094.1	_	metallophosphatase	6.9	1.0	NF	0.1	IM	CY	GO:0005886	P
282	WP_000111455.1	LIC_11417	MULTISPECIES: ATP-dependent Clp protease proteolytic subunit	128.0	16.9	1.8	0.1	CY	CY	GO:0005886	NP
283	WP_000478209.1	LIC_10001	chromosomal replication initiator protein DnaA	6.5	0.9	NF	0.1	CY	CY	GO:0005737	NP
284	WP_000821095.1	LIC_10302	hypothetical protein	82.0	NF	11.8	0.1	UN	OM	GO:0005615	P
285	WP_000854082.1	_	hypothetical protein	26.1	3.7	NF	0.1	CY	CY	GO:0005886	NP
286	WP_000488503.1	LIC_10314	hypothetical protein	440.7	56.6	6.3	0.1	UN	OM	GO:0005615	P
287	WP_001247263.1	LIC_12328	VOC family protein	624.9	78.1	10.1	0.1	CY	CY	GO:0005737	P
288	WP_002070138.1	_	glucanase	91.5	12.8	NF	0.1	UN	OM	GO:0005737	P
289	WP_000692396.1	LIC_10361	electron transfer flavoprotein subunit beta	3198.8	62.1	383.9	0.1	CY	CY	GO:0005737	NP
290	WP_001206796.1	_	MULTISPECIES: 50S ribosomal protein L33	368.6	NF	51.3	0.1	UN	CY	GO:0005737	NP
291	WP_001058137.1	LIC_11809	monothiol glutaredoxin, Grx4 family	104.4	13.1	1.4	0.1	UN	CY	GO:0005737	NP
292	WP_000004519.1	LIC_11913	lysine--tRNA ligase	154.3	0.8	20.6	0.1	CY	CY	GO:0005737	NP
293	WP_000424716.1	_	polyamine aminopropyltransferase 2	478.0	5.3	60.9	0.1	CY	CY	GO:0005737	NP
294	WP_000096242.1	_	hypothetical protein	9.1	0.2	1.0	0.1	UN	OM	GO:0005615	P
295	WP_000995952.1	LIC_11767	MULTISPECIES: aspartate aminotransferase family protein	159.5	1.8	20.1	0.1	CY	CY	GO:0005737	NP
296	WP_000134482.1	LIC_11136	transcriptional regulator	17.1	NF	2.3	0.1	CY	CY	GO:0005737	NP

297	WP_001071872.1	LIC_12675	DUF4303 domain-containing protein	13.2	0.0	1.8	0.1	CY	CY	GO:0005737	NP
298	WP_000125234.1	LIC_20143	serine protease	135.8	7.9	10.5	0.1	UN	OM	GO:0005737	NP
299	WP_000431287.1	LIC_10048	histidine--tRNA ligase	41.1	0.9	4.6	0.1	CY	CY	GO:0005737	NP
300	WP_001053134.1	LIC_12871	50S ribosomal protein L23	578.9	NF	77.7	0.1	UN	CY	GO:0005737	NP
301	WP_000039671.1	LIC_11380	MULTISPECIES: RNA polymerase sigma factor WhiG	249.2	NF	33.4	0.1	CY	CY	GO:0005737	NP
302	WP_000406878.1	LIC_11004	MULTISPECIES: anti-sigma factor antagonist	986.7	106.5	25.0	0.1	UN	CY	GO:0005737	NP
303	WP_000454661.1	LIC_11406	MULTISPECIES: hypothetical protein	310.3	NF	41.2	0.1	UN	CY	GO:0019867	NP
304	WP_001284494.1	_	MULTISPECIES: transcriptional regulator	109.0	4.4	10.0	0.1	UN	CY	GO:0005737	P
305	WP_000041092.1	LIC_11194	aldolase	3488.0	213.0	249.4	0.1	CY	CY	GO:0005737	NP
306	WP_000072965.1	LIC_10360	electron transfer flavoprotein subunit alpha/FixB family protein	1752.5	6.9	225.0	0.1	UN	CY	GO:0005737	NP
307	WP_000043153.1	_	short-chain dehydrogenase	767.9	36.7	64.6	0.1	CY	PP	GO:0005737	NP
308	WP_000406975.1	_	MULTISPECIES: anti-sigma factor antagonist	514.8	39.1	28.6	0.1	CY	CY	GO:0005737	NP
309	WP_000918539.1	_	N-acetylmuramoyl-L-alanine amidase	3.1	0.4	NF	0.1	UN	CY	GO:0005737	NP
310	WP_001274139.1	LIC_13009	acyl-CoA dehydrogenase	2257.1	17.2	278.3	0.1	CY	CY	GO:0005737	NP
311	WP_001077104.1	LIC_11203	signal transduction protein	16.2	NF	2.1	0.1	IM	CY	GO:0005737	NP
312	WP_000739193.1	LIC_20099	shikimate kinase	43.1	NF	5.6	0.1	CY	CY	GO:0005737	NP
313	WP_000115097.1	LIC_10761	50S ribosomal protein L13	663.0	0.8	84.7	0.1	CY	PP	GO:0005737	NP
314	WP_000356374.1	LIC_12504	MULTISPECIES: response regulator	78.1	3.0	7.0	0.1	CY	CY	GO:0005737	NP

315	WP_001294723.1	LIC_12970	cell division protein FtsA	110.6	2.0	12.1	0.1	CY	CY	GO:0005737	NP
316	WP_000454163.1	LIC_11641	radical SAM protein	62.6	NF	8.0	0.1	CY	CY	GO:0005737	NP
317	WP_000249791.1	LIC_11725	MULTISPECIES: glyoxalase/bleomycin resistance/dioxygenase family protein	15.9	2.0	NF	0.1	UN	CY	GO:0005737	P
318	WP_000379289.1	LIC_10808	PaaI family thioesterase	161.6	NF	20.5	0.1	CY	CY	GO:0005737	NP
319	WP_000666507.1	LIC_10684	cytochrome c	49.9	6.3	NF	0.1	UN	PP	GO:0005615	NP
320	WP_000455718.1	LIC_12994	DUF1577 domain-containing protein	50.8	NF	6.4	0.1	CY	CY	GO:0005737	NP
321	WP_001284168.1	LIC_12841	LL-diaminopimelate aminotransferase	622.2	19.8	57.9	0.1	CY	OM	GO:0005737	NP
322	WP_001079022.1	LIC_20152	hypothetical protein	8.6	1.1	NF	0.1	UN	EC	GO:0005615	P
323	WP_000776292.1	LIC_12458	5-formyltetrahydrofolate cyclo-ligase	8.4	1.0	NF	0.1	CY	CY	GO:0005737	NP
324	WP_000239305.1	LIC_10191	OmpA family protein	6913.4	657.9	192.2	0.1	UN	PP	GO:0005615	P
325	WP_002070132.1	_	glycerophosphoryl diester phosphodiesterase	17.2	NF	2.1	0.1	CY	CY	GO:0005886	NP
326	WP_000735725.1	LIC_20061	tetratricopeptide repeat protein	13.8	1.7	NF	0.1	CY	OM	GO:0005737	NP
327	WP_000403254.1	LIC_12782	hydroxymethylglutaryl-CoA lyase	38.2	NF	4.6	0.1	CY	CY	GO:0005737	NP
328	WP_000959560.1	LIC_13155	Hpt domain-containing protein	130.4	15.5	NF	0.1	UN	CY	GO:0005737	NP
329	WP_000433142.1	LIC_10279	hypothetical protein	4.7	NF	0.6	0.1	CY	CY	GO:0005737	NP
330	WP_000678902.1	LIC_13050	hypothetical protein	1067.1	112.1	13.7	0.1	UN	EC	GO:0005615	P
331	WP_000603580.1	LIC_12731	hypothetical protein	44.5	5.2	NF	0.1	UN	OM	GO:0005737	P
332	WP_000769736.1	LIC_12287	hypothetical protein	141.3	NF	16.4	0.1	UN	PP	GO:0005615	NP

333	WP_000799870.1	LIC_13436	hypothetical protein	35.2	4.1	NF	0.1	UN	OM	GO:0005615	P
334	WP_001068357.1	LIC_13042	N-acetyltransferase	37.6	4.4	NF	0.1	UN	CY	GO:0005737	P
335	WP_000886261.1	LIC_13480	serine--tRNA ligase	152.0	0.9	16.7	0.1	CY	CY	GO:0005737	NP
336	WP_000562954.1	LIC_10984	hypothetical protein	227.5	26.2	NF	0.1	CY	PP	GO:0005737	NP
337	WP_000540770.1	_	NADH-quinone oxidoreductase subunit NuoE	69.3	NF	7.9	0.1	CY	CY	GO:0005737	NP
338	WP_000670745.1	_	hypothetical protein	25.4	2.9	NF	0.1	UN	CY	GO:0005886	P
339	WP_000740026.1	LIC_20215	hypothetical protein	9.5	1.1	NF	0.1	UN	EC	GO:0005737	P
340	WP_000282526.1	_	response regulator	52.9	6.0	NF	0.1	CY	CY	GO:0005737	NP
341	WP_001015936.1	LIC_11739	peptidase inhibitor	59.7	6.7	NF	0.1	UN	OM	GO:0005886	P
342	WP_000562102.1	LIC_12702	MULTISPECIES: 30S ribosomal protein S15	782.8	NF	87.1	0.1	CY	CY	GO:0005737	NP
343	WP_000849259.1	_	MULTISPECIES: DNA-binding response regulator	340.9	37.5	0.3	0.1	CY	CY	GO:0005737	NP
344	WP_000683520.1	LIC_12108	aspartate--tRNA ligase	348.4	3.1	35.4	0.1	CY	CY	GO:0005737	NP
345	WP_000615621.1	_	protein translocase subunit SecA	156.5	0.7	16.5	0.1	CY	CY	GO:0005737	NP
346	WP_001151672.1	LIC_12739	MaoC family dehydratase	945.0	64.6	38.5	0.1	CY	CY	GO:0005737	NP
347	WP_000604760.1	_	hypothetical protein	121.5	13.1	NF	0.1	UN	OM	GO:0005737	P
348	WP_000510649.1	LIC_13449	flagellar assembly protein FliW	157.2	16.8	NF	0.1	IM	CY	GO:0005737	P
349	WP_001246923.1	LIC_12113	MULTISPECIES: 30S ribosomal protein S6	374.3	NF	39.6	0.1	UN	CY	GO:0005737	NP
350	WP_000102400.1	LIC_10752	50S ribosomal protein L7/L12	5175.0	476.1	66.7	0.1	UN	CY	GO:0005737	NP

351	WP_001284348.1	LIC_13393	ketol-acid reductoisomerase	1827.2	46.2	145.1	0.1	CY	CY	GO:0005737	NP
352	WP_001190627.1	LIC_12618	diaminopimelate decarboxylase	389.6	15.3	25.3	0.1	CY	OM	GO:0005737	NP
353	WP_000031821.1	LIC_10524	molecular chaperone DnaK	4128.8	390.3	39.6	0.1	CY	CY	GO:0005737	NP
354	WP_001190664.1	LIC_20204	aminotransferase class V-fold PLP-dependent enzyme	29.6	3.1	NF	0.1	CY	CY	GO:0005737	NP
355	WP_000567237.1	LIC_11016	MULTISPECIES: adenylosuccinate lyase	204.7	21.2	NF	0.1	CY	CY	GO:0005737	NP
356	WP_000693600.1	LIC_11267	sulfate ABC transporter substrate-binding protein	487.6	50.4	NF	0.1	PP	PP	GO:0005615	NP
357	WP_000233562.1	LIC_13223	inorganic pyrophosphatase	284.3	26.1	3.3	0.1	CY	CY	GO:0005737	NP
358	WP_000400940.1	LIC_10029	inositol monophosphatase	89.8	2.7	6.4	0.1	CY	CY	GO:0005737	NP
359	WP_000869002.1	LIC_10791	cAMP-binding protein	105.6	NF	10.7	0.1	CY	CY	GO:0005737	NP
360	WP_000828548.1	LIC_20209	methylmalonyl-CoA mutase	97.9	9.7	0.2	0.1	CY	CY	GO:0005737	NP
361	WP_000067921.1	LIC_11462	imidazole glycerol phosphate synthase cyclase subunit	43.9	NF	4.4	0.1	CY	CY	GO:0005737	NP
362	WP_000227049.1	LIC_11115	thiosulfate sulfurtransferase	660.0	54.8	10.6	0.1	CY	CY	GO:0005737	NP
363	WP_000764038.1	_	MULTISPECIES: ferredoxin	279.0	27.6	NF	0.1	UN	CY	GO:0005737	NP
364	WP_000339988.1	LIC_10138	hypothetical protein	54.1	4.4	0.9	0.1	CY	CY	GO:0005737	NP
365	WP_001011583.1	LIC_10955	4-hydroxy-3-methylbut-2-en-1-yl diphosphate synthase (flavodoxin)	73.3	NF	7.2	0.1	CY	OM	GO:0005737	NP
366	WP_000538752.1	LIC_10607	DegT/DnrJ/EryC1/StrS family aminotransferase	463.5	35.3	10.3	0.1	CY	CY	GO:0005737	NP

367	WP_001072660.1	LIC_11559	50S ribosomal protein L19	1688.7	14.2	151.4	0.1	CY	CY	GO:0005737	NP
368	WP_000172195.1	LIC_20228	elongation factor P	476.1	16.8	29.8	0.1	CY	CY	GO:0005737	NP
369	WP_000664814.1	LIC_12944	30S ribosomal protein S1	163.5	1.6	14.4	0.1	CY	CY	GO:0005737	NP
370	WP_000282348.1	LIC_12098	MULTISPECIES: hypothetical protein	6.9	NF	0.7	0.1	CY	CY	GO:0005737	NP
371	WP_000081487.1	LIC_10430	MULTISPECIES: 50S ribosomal protein L25	217.6	NF	21.0	0.1	CY	CY	GO:0005737	NP
372	WP_000393538.1	_	acyl-CoA dehydrogenase	854.1	8.3	73.8	0.1	CY	CY	GO:0005737	NP
373	WP_001125898.1	LIC_12488	acyl-CoA thioesterase	66.8	4.4	2.0	0.1	CY	EC	GO:0005737	NP
374	WP_000351733.1	LIC_11825	phosphoheptose isomerase	131.1	3.5	9.1	0.1	CY	CY	GO:0005737	NP
375	WP_000575569.1	LIC_11258	MULTISPECIES: rod shape-determining protein	1585.1	1.4	148.9	0.1	CY	CY	GO:0005737	NP
376	WP_000149893.1	LIC_12701	polyribonucleotide nucleotidyltransferase	727.3	50.8	18.0	0.1	CY	CY	GO:0005737	NP
377	WP_000053346.1	LIC_11354	S-adenosylmethionine synthase	1579.6	4.7	143.3	0.1	CY	CY	GO:0005737	NP
378	WP_000004530.1	LIC_11195	ornithine carbamoyltransferase	144.9	12.4	1.2	0.1	CY	CY	GO:0005737	NP
379	WP_000033675.1	LIC_10771	O-methyltransferase	7.9	0.7	NF	0.1	CY	CY	GO:0005737	NP
380	WP_000488432.1	LIC_11536	NlpC/P60 family protein	39.4	3.2	0.4	0.1	UN	OM	GO:0005615	P
381	WP_082274816.1	_	guanylate kinase	22.9	2.1	NF	0.1	CY	CY	GO:0005737	NP
382	WP_000945708.1	_	hypothetical protein	28.7	2.1	0.5	0.1	UN	OM	GO:0005615	P
383	WP_000665700.1	LIC_10996	signal transduction protein	9.2	NF	0.8	0.1	IM	CY	GO:0005737	NP
384	WP_000852369.1	LIC_10310	glycine cleavage system protein H	467.7	41.9	NF	0.1	UN	CY	GO:0005737	NP

385	WP_000809310.1	LIC_11882	DUF2797 domain-containing protein	32.8	NF	2.9	0.1	CY	CY	GO:0005737	NP
386	WP_000701966.1	LIC_11409	acetolactate synthase, large subunit, biosynthetic type	159.7	0.2	14.1	0.1	CY	CY	GO:0005737	NP
387	WP_001969787.1	LIC_12016	transcriptional regulator	19.6	NF	1.7	0.1	UN	CY	GO:0005737	NP
388	WP_001070308.1	LIC_12026	MULTISPECIES: bifunctional methylenetetrahydrofolate dehydrogenase/methenyltetrahydrofolate cyclohydrolase	129.3	1.3	10.1	0.1	CY	CY	GO:0005737	NP
389	WP_000432947.1	LIC_12165	radical SAM protein	430.1	0.5	37.3	0.1	CY	CY	GO:0005737	NP
390	WP_001014491.1	LIC_13055	hypothetical protein	17.5	1.5	NF	0.1	UN	OM	GO:0005615	P
391	WP_000935311.1	_	DUF1343 domain-containing protein	92.7	6.8	1.3	0.1	PP	CY	GO:0005737	NP
392	WP_000636970.1	LIC_12478	MBL fold metallo-hydrolase	46.3	NF	4.1	0.1	CY	CY	GO:0005737	P
393	WP_001974549.1	LIC_11198	guanylate cyclase	1189.2	2.6	101.6	0.1	CY	CY	GO:0005737	NP
394	WP_000998676.1	LIC_10019	NAD(P)-dependent oxidoreductase	93.4	NF	8.2	0.1	CY	CY	GO:0005737	NP
395	WP_001136732.1	LIC_11219	peroxiredoxin	4298.3	196.5	177.7	0.1	CY	CY	GO:0005737	P
396	WP_000204200.1	LIC_11479	MULTISPECIES: tyrosine recombinase XerD	106.1	3.5	5.7	0.1	CY	CY	GO:0005737	NP
397	WP_000004280.1	_	hypothetical protein	194.9	16.9	NF	0.1	CY	CY	GO:0005737	NP
398	WP_000876417.1	LIC_11436	sigma factor regulatory protein FecR	53.2	4.6	NF	0.1	OM	OM	GO:0005886	NP
399	WP_000188441.1	LIC_11110	two-component system response regulator	89.7	NF	7.7	0.1	CY	CY	GO:0005737	NP
400	WP_000418124.1	_	MULTISPECIES: XRE family transcriptional regulator	21.8	1.9	NF	0.1	CY	CY	GO:0005737	NP

401	WP_001023250.1	LIC_13094	5'-nucleotidase SurE	73.8	6.4	NF	0.1	CY	EC	GO:0005737	NP
402	WP_000475904.1	LIC_12141	KR domain-containing protein	160.5	0.2	13.6	0.1	CY	CY	GO:0005737	NP
403	WP_000355766.1	LIC_13162	3-deoxy-D-arabino-heptulosonate 7-phosphate synthase	656.2	8.0	48.4	0.1	CY	CY	GO:0005737	NP
404	WP_001053431.1	LIC_12995	hypothetical protein	12.1	NF	1.0	0.1	UN	CY	GO:0005737	NP
405	WP_000145482.1	_	phosphohistidine phosphatase	15.3	0.8	0.5	0.1	CY	CY	GO:0005737	NP
406	WP_000135448.1	LIC_10853	MULTISPECIES: ribosome-recycling factor	657.2	53.3	2.9	0.1	CY	CY	GO:0005737	NP
407	WP_000712192.1	LIC_12854	50S ribosomal protein L15	1732.7	11.8	135.9	0.1	CY	PP	GO:0005737	NP
408	WP_000671777.1	LIC_13397	phosphodiesterase	22.9	1.9	NF	0.1	EC	OM	GO:0005615	P
409	WP_000201440.1	LIC_20227	acireductone dioxygenase	791.2	56.5	8.9	0.1	CY	CY	GO:0005737	NP
410	WP_000815636.1	_	antibiotic biosynthesis monooxygenase	118.4	9.8	NF	0.1	UN	CY	GO:0005737	NP
411	WP_000111480.1	LIC_10850	30S ribosomal protein S2	384.8	3.2	28.5	0.1	CY	CY	GO:0005737	NP
412	WP_000702258.1	LIC_10499	DUF1566 domain-containing protein	21.3	1.7	NF	0.1	UN	EC	GO:0005615	P
413	WP_000031888.1	LIC_11617	MULTISPECIES: ArsR family transcriptional regulator	5445.1	131.8	313.3	0.1	UN	CY	GO:0005737	NP
414	WP_000450144.1	LIC_20032	hypothetical protein	99.6	8.1	NF	0.1	CY	CY	GO:0005737	NP
415	WP_000557278.1	LIC_10537	membrane protein	4.1	0.3	NF	0.1	OM	OM	GO:0005615	P
416	WP_000672174.1	LIC_12641	hypothetical protein	17.6	0.7	0.7	0.1	OM	OM	GO:0005615	P
417	WP_001189133.1	LIC_12463	MULTISPECIES: translation initiation factor IF-3	131.0	1.2	9.4	0.1	CY	CY	GO:0005737	NP

418	WP_000132016.1	LIC_12177	acylneuraminate cytidylyltransferase family protein	37.7	0.1	2.9	0.1	CY	CY	GO:0005737	NP
419	WP_001124477.1	_	TetR/AcrR family transcriptional regulator	66.0	NF	5.3	0.1	CY	CY	GO:0005737	NP
420	WP_000845534.1	LIC_12637	MULTISPECIES: 50S ribosomal protein L31	125.5	3.0	7.0	0.1	CY	CY	GO:0005737	NP
421	WP_001054393.1	LIC_20047	DUF839 domain-containing protein	11.8	0.0	0.9	0.1	UN	PP	GO:0005737	NP
422	WP_000088214.1	LIC_12032	catalase	693.8	32.2	22.8	0.1	PP	PP	GO:0005737	NP
423	WP_001982280.1	LIC_12839	aspartate carbamoyltransferase	144.2	3.6	7.7	0.1	CY	CY	GO:0005737	NP
424	WP_000119461.1	LIC_13297	MULTISPECIES: hypothetical protein	1200.3	0.1	94.1	0.1	UN	CY	GO:0005737	NP
425	WP_000048017.1	LIC_11781	malate dehydrogenase	2346.7	123.5	60.2	0.1	UN	CY	GO:0005737	NP
426	WP_001051846.1	LIC_12873	50S ribosomal protein L3	627.0	NF	49.1	0.1	CY	PP	GO:0005737	NP
427	WP_000907506.1	LIC_12619	hypothetical protein	5.6	NF	0.4	0.1	CY	CY	GO:0005737	P
428	WP_000849962.1	LIC_11812	MULTISPECIES: glutamate--cysteine ligase	69.5	1.3	4.1	0.1	CY	CY	GO:0005737	NP
429	WP_001211488.1	_	hypothetical protein	25.8	NF	2.0	0.1	UN	CY	GO:0005737	P
430	WP_000708723.1	LIC_11534	ATP:cob(I)alamin adenosyltransferase	86.1	1.3	5.4	0.1	CY	CY	GO:0005737	NP
431	WP_000389996.1	LIC_10838	VOC family protein	491.3	28.7	9.2	0.1	CY	CY	GO:0005737	P
432	WP_001173977.1	LIC_12040	MULTISPECIES: VOC family protein	377.2	29.1	NF	0.1	UN	CY	GO:0005737	P
433	WP_000277167.1	LIC_13276	hypothetical protein	77.5	6.0	NF	0.1	UN	CY	GO:0005737	NP
434	WP_002074815.1	_	hypothetical protein	19.7	1.5	NF	0.1	UN	PP	GO:0005615	P
435	WP_000754808.1	LIC_10672	hypothetical protein	674.9	45.9	5.5	0.1	UN	CY	GO:0005886	P

436	WP_000797254.1	LIC_11250	hypothetical protein	11.4	0.9	NF	0.1	UN	CY	GO:0005737	NP
437	WP_001131967.1	_	hypothetical protein	208.2	2.9	12.8	0.1	CY	CY	GO:0005737	NP
438	WP_000060053.1	LIC_11196	hypothetical protein	615.7	42.0	4.3	0.1	UN	CY	GO:0019867	NP
439	WP_000113840.1	LIC_11760	MULTISPECIES: 50S ribosomal protein L28	1536.3	2.0	113.2	0.1	CY	CY	GO:0005737	NP
440	WP_001001943.1	LIC_10066	enoyl-CoA hydratase	67.3	NF	5.0	0.1	CY	CY	GO:0005737	NP
441	WP_001246618.1	LIC_12194	transketolase	460.0	3.3	31.1	0.1	CY	CY	GO:0005737	NP
442	WP_000390685.1	LIC_13405	GTP cyclohydrolase I FolE	168.1	2.1	10.5	0.1	CY	CY	GO:0005737	NP
443	WP_000348634.1	LIC_10533	ABC transporter substrate-binding protein	7.6	0.6	NF	0.1	UN	CY	GO:0005886	P
444	WP_001022430.1	LIC_20205	rubrerythrin	495.8	14.2	22.7	0.1	UN	PP	GO:0005737	P
445	WP_000179609.1	LIC_12636	transcription termination factor Rho	378.5	3.1	25.1	0.1	CY	CY	GO:0005737	NP
446	WP_000828653.1	LIC_10549	non-canonical purine NTP pyrophosphatase	43.2	NF	3.2	0.1	CY	CY	GO:0005737	NP
447	WP_000667225.1	LIC_11608	hypothetical protein	103.5	1.9	5.8	0.1	CY	CY	GO:0005737	NP
448	WP_000719443.1	LIC_10317	ribose 5-phosphate isomerase B	111.7	3.1	5.1	0.1	CY	CY	GO:0005737	NP
449	WP_001191099.1	LIC_11811	bifunctional glutamate--cysteine ligase GshA/glutathione synthetase GshB	24.2	NF	1.8	0.1	CY	CY	GO:0005737	NP
450	WP_001232578.1	LIC_12094	triose-phosphate isomerase	345.0	7.5	17.8	0.1	IM	OM	GO:0005737	NP
451	WP_001070780.1	LIC_12243	mannose-1-phosphate guanylyltransferase	155.0	11.3	NF	0.1	CY	CY	GO:0005737	NP
452	WP_000948326.1	LIC_10578	hypothetical protein	184.2	2.8	10.6	0.1	CY	OM	GO:0005886	P
453	WP_000856053.1	LIC_11832	NADPH-dependent 7-cyano-7-deazaguanine reductase QueF	786.4	39.0	18.4	0.1	CY	CY	GO:0005737	NP

454	WP_000032804.1	LIC_11496	anti-sigma factor antagonist	49.6	3.6	NF	0.1	CY	CY	GO:0019867	NP
455	WP_000366324.1	LIC_13237	TIGR00730 family Rossman fold protein	19.6	NF	1.4	0.1	CY	CY	GO:0005737	NP
456	WP_000101124.1	LIC_12454	MULTISPECIES: response regulator	1635.0	117.7	NF	0.1	CY	CY	GO:0005737	NP
457	WP_000432936.1	LIC_10158	RNA-binding protein	458.6	3.4	29.5	0.1	CY	CY	GO:0005737	NP
458	WP_001201672.1	LIC_11140	alpha-galactosidase	101.1	7.2	NF	0.1	CY	CY	GO:0005737	P
459	WP_001075198.1	LIC_12564	ATP phosphoribosyltransferase regulatory subunit	85.8	6.0	NF	0.1	CY	OM	GO:0005737	NP
460	WP_000723997.1	LIC_12539	hypothetical protein	249.7	11.5	6.1	0.1	EC	OM	GO:0005615	P
461	WP_001253859.1	LIC_11038	pyridoxal phosphate-dependent aminotransferase	24.8	0.3	1.4	0.1	CY	CY	GO:0005737	NP
462	WP_000123883.1	LIC_12864	MULTISPECIES: 30S ribosomal protein S17	1003.7	3.4	66.8	0.1	CY	CY	GO:0005737	NP
463	WP_000799846.1	LIC_11768	3-isopropylmalate dehydrogenase	317.1	22.1	NF	0.1	CY	CY	GO:0005737	NP
464	WP_001280808.1	LIC_13301	MULTISPECIES: acetyl-CoA acetyltransferase	1349.7	22.9	71.3	0.1	UN	CY	GO:0005737	NP
465	WP_001166258.1	LIC_11983	MULTISPECIES: hypothetical protein	63.7	4.4	NF	0.1	CY	OM	GO:0005737	P
466	WP_000215708.1	LIC_11302	HAD family hydrolase	42.1	2.9	NF	0.1	CY	CY	GO:0005737	NP
467	WP_000690880.1	LIC_13154	acyl-ACP--UDP-N-acetylglucosamine O-acyltransferase	537.9	20.6	16.8	0.1	CY	CY	GO:0005737	NP
468	WP_001982628.1	_	MULTISPECIES: acetylglutamate kinase	28.4	NF	2.0	0.1	CY	CY	GO:0005737	NP
469	WP_001128627.1	LIC_11141	MULTISPECIES: ribonuclease D	216.5	13.6	1.2	0.1	CY	CY	GO:0005737	NP
470	WP_000182263.1	LIC_12447	30S ribosomal protein S1	1115.1	15.5	60.9	0.1	CY	CY	GO:0005737	NP

471	WP_000339879.1	LIC_12591	aminopeptidase N	310.4	18.4	2.6	0.1	CY	CY	GO:0005737	NP
472	WP_000086941.1	LIC_13262	amidophosphoribosyltransferase	455.4	3.7	27.0	0.1	CY	CY	GO:0005737	NP
473	WP_000683647.1	LIC_11051	WGR domain-containing protein	25.1	0.4	1.2	0.1	EC	OM	GO:0005737	P
474	WP_001226846.1	LIC_20153	hypothetical protein	161.7	10.4	0.2	0.1	UN	EC	GO:0005737	P
475	WP_000266284.1	_	MULTISPECIES: hypothetical protein	51.1	0.8	2.6	0.1	UN	CY	GO:0005737	P
476	WP_000741905.1	LIC_10851	elongation factor Ts	910.7	43.5	16.1	0.1	CY	CY	GO:0005737	NP
477	WP_000712870.1	LIC_11563	metal-dependent phosphohydrolase	39.2	NF	2.6	0.1	CY	CY	GO:0005737	NP
478	WP_000431183.1	LIC_11408	hypothetical protein	215.9	7.1	7.0	0.1	CY	CY	GO:0005737	P
479	WP_001280999.1	LIC_12010	elongation factor 4	91.2	0.3	5.6	0.1	IM	CY	GO:0005737	NP
480	WP_000654116.1	LIC_12305	LexA repressor	32.8	NF	2.1	0.1	CY	CY	GO:0005737	NP
481	WP_000475809.1	LIC_13123	MULTISPECIES: hypothetical protein	334.1	1.9	19.7	0.1	UN	CY	GO:0005615	NP
482	WP_000387530.1	LIC_12868	MULTISPECIES: 50S ribosomal protein L22	1199.8	NF	77.5	0.1	CY	CY	GO:0005737	NP
483	WP_000433680.1	LIC_12541	MULTISPECIES: anthranilate phosphoribosyltransferase	105.0	5.5	1.3	0.1	CY	CY	GO:0005737	NP
484	WP_000846752.1	LIC_11137	hypothetical protein	18.5	NF	1.2	0.1	UN	CY	GO:0005737	NP
485	WP_001292536.1	LIC_10732	thioredoxin-dependent thiol peroxidase	442.7	14.6	13.8	0.1	CY	CY	GO:0005737	P
486	WP_000365890.1	LIC_11790	pyridoxal phosphate-dependent aminotransferase	347.7	14.1	8.3	0.1	CY	OM	GO:0005737	NP
487	WP_001198107.1	LIC_12198	DegT/DnrJ/EryC1/StrS family aminotransferase	488.5	5.6	25.7	0.1	CY	CY	GO:0005737	NP

488	WP_001283275.1	LIC_10764	YajQ family cyclic di-GMP-binding protein	157.2	4.8	5.3	0.1	CY	CY	GO:0005737	NP
489	WP_001092943.1	LIC_10446	phosphoglucosamine mutase	15.1	0.2	0.8	0.1	CY	OM	GO:0005737	NP
490	WP_000013232.1	LIC_20208	methylmalonyl-CoA mutase	100.1	4.3	2.0	0.1	CY	CY	GO:0005737	NP
491	WP_000517242.1	_	class I SAM-dependent methyltransferase	176.8	0.4	10.9	0.1	CY	CY	GO:0005737	NP
492	WP_000054108.1	LIC_12846	MULTISPECIES: DNA-directed RNA polymerase subunit alpha	2259.6	41.0	101.7	0.1	CY	CY	GO:0005737	NP
493	WP_000914951.1	LIC_13434	hypothetical protein	1378.2	18.8	68.0	0.1	UN	OM	GO:0005886	P
494	WP_000117570.1	LIC_20083	adenosylhomocysteinase	2500.0	18.5	138.5	0.1	CY	CY	GO:0005737	NP
495	WP_000161597.1	LIC_11359	acyl dehydratase	647.6	39.4	1.2	0.1	CY	CY	GO:0005886	NP
496	WP_000898172.1	LIC_13281	acyl-CoA dehydrogenase	24.9	NF	1.6	0.1	CY	CY	GO:0005886	NP
497	WP_000523717.1	LIC_11247	hypothetical protein	19.5	NF	1.2	0.1	CY	CY	GO:0005737	P
498	WP_001122022.1	LIC_11070	iron-containing alcohol dehydrogenase	64.6	NF	3.9	0.1	CY	CY	GO:0005737	NP
499	WP_000775825.1	LIC_11848	hypothetical protein	2311.3	95.0	46.0	0.1	UN	PP	GO:0005615	NP
500	WP_000850434.1	_	hypothetical protein	27.7	1.7	NF	0.1	UN	CY	GO:0005737	P
501	WP_001078417.1	LIC_11006	transcriptional repressor	296.0	0.6	17.3	0.1	CY	CY	GO:0005737	NP
502	WP_000762669.1	LIC_12017	chaperone protein ClpB	177.3	9.1	1.6	0.1	CY	CY	GO:0005737	NP
503	WP_001011233.1	LIC_10831	hypothetical protein	2.4	0.1	NF	0.1	EC	EC	GO:0005737	P
504	WP_000647839.1	LIC_10858	proline--tRNA ligase	175.2	1.7	8.7	0.1	CY	CY	GO:0005737	NP
505	WP_001110736.1	LIC_10723	flagellar hook protein FliD	8.3	0.5	NF	0.1	PP	EC	GO:0005737	P

506	WP_000990075.1	LIC_12744	NADH-quinone oxidoreductase subunit D	513.8	2.3	28.1	0.1	CY	CY	GO:0005737	NP
507	WP_001271670.1	LIC_12475	dihydrolipoyl dehydrogenase	440.2	24.9	1.1	0.1	CY	CY	GO:0005737	NP
508	WP_000956489.1	_	ATP phosphoribosyltransferase	146.0	8.6	NF	0.1	CY	CY	GO:0005737	NP
509	WP_000410308.1	_	1-acyl-sn-glycerol-3-phosphate acyltransferase	63.9	NF	3.8	0.1	UN	CY	GO:0005886	NP
510	WP_000676629.1	LIC_20054	DNA-binding protein	26.8	0.3	1.3	0.1	UN	CY	GO:0005886	NP
511	WP_000643584.1	LIC_11769	MULTISPECIES: response regulator	226.4	10.5	2.8	0.1	CY	CY	GO:0005737	NP
512	WP_000181059.1	LIC_11309	RNA methyltransferase	51.5	0.5	2.5	0.1	CY	CY	GO:0005737	NP
513	WP_000439024.1	LIC_12452	SMC-Scp complex subunit ScpB	63.3	NF	3.7	0.1	CY	CY	GO:0005737	NP
514	WP_000622941.1	LIC_10406	hypothetical protein	20.5	NF	1.2	0.1	CY	CY	GO:0005737	P
515	WP_000857390.1	LIC_10162	class II fumarate hydratase	398.6	23.2	NF	0.1	CY	CY	GO:0005737	NP
516	WP_000928025.1	LIC_12792	MULTISPECIES: type III pantothenate kinase	50.8	0.9	2.1	0.1	CY	IM	GO:0005737	NP
517	WP_000695822.1	LIC_10002	DNA polymerase III subunit beta	970.3	54.2	2.3	0.1	CY	CY	GO:0005737	NP
518	WP_000877551.1	LIC_11729	NADPH-dependent 2,4-dienoyl-CoA reductase	190.7	NF	11.0	0.1	CY	CY	GO:0005737	NP
519	WP_000646252.1	LIC_12202	NAD dependent epimerase/dehydratase	633.1	4.0	32.5	0.1	CY	CY	GO:0005737	NP
520	WP_000802062.1	_	MULTISPECIES: endoflagellar motor protein	375.2	21.6	NF	0.1	UN	CY	GO:0005615	P
521	WP_000863312.1	LIC_20249	aconitate hydratase	723.3	35.4	6.1	0.1	CY	PP	GO:0005737	NP
522	WP_000626143.1	LIC_10538	MULTISPECIES: ABC-F family ATPase	240.7	0.3	13.5	0.1	CY	CY	GO:0005737	P

523	WP_000622506.1	LIC_10847	MULTISPECIES: tetratricopeptide repeat protein	113.1	6.4	NF	0.1	UN	CY	GO:0005737	P
524	WP_000017889.1	LIC_11874	hypothetical protein	57.9	NF	3.3	0.1	CY	OM	GO:0005615	P
525	WP_001970781.1	_	4-hydroxy-tetrahydrodipicolinate synthase	325.2	17.3	1.1	0.1	CY	CY	GO:0005737	NP
526	WP_000172862.1	_	hypothetical protein	23.2	0.8	0.5	0.1	CY	CY	GO:0005737	NP
527	WP_000091728.1	LIC_11540	CTP synthetase	249.1	NF	14.1	0.1	CY	CY	GO:0005737	NP
528	WP_000604072.1	LIC_10065	deoxycytidine triphosphate deaminase	795.2	29.8	15.1	0.1	CY	CY	GO:0005737	NP
529	WP_001971016.1	_	hypothetical protein	245.1	13.8	NF	0.1	UN	EC	GO:0005886	P
530	WP_001274661.1	LIC_10601	MaoC family dehydratase	696.8	16.2	22.9	0.1	CY	CY	GO:0005737	NP
531	WP_000068289.1	LIC_13324	argininosuccinate synthase	366.7	8.7	11.8	0.1	CY	CY	GO:0005737	NP
532	WP_000702812.1	LIC_11554	ribulose-phosphate 3-epimerase	124.3	6.9	NF	0.1	CY	CY	GO:0005737	NP
533	WP_000572205.1	LIC_10041	transcriptional antiterminator	50.8	0.4	2.4	0.1	CY	CY	GO:0005737	NP
534	WP_000126723.1	LIC_10918	activator of HSP90 ATPase	516.4	28.4	NF	0.1	CY	CY	GO:0005737	NP
535	WP_000187969.1	_	sulfurtransferase	27.3	NF	1.5	0.1	UN	CY	GO:0005737	NP
536	WP_002084923.1	LIC_10383	2,3-bisphosphoglycerate-independent phosphoglycerate mutase	181.6	9.9	NF	0.1	CY	PP	GO:0005737	NP
537	WP_002079862.1	_	hypothetical protein	41.2	2.3	NF	0.1	UN	OM	GO:0005886	P
538	WP_002082140.1	LIC_12164	class I SAM-dependent methyltransferase	67.0	NF	3.7	0.1	CY	CY	GO:0005737	NP
539	WP_000374062.1	LIC_11918	outer membrane lipoprotein-sorting protein	13.4	0.7	NF	0.1	UN	OM	GO:0005615	NP
540	WP_000063951.1	LIC_11405	helicase	336.0	18.3	NF	0.1	CY	CY	GO:0005737	NP

541	WP_000662272.1	LIC_10759	thiamine-monophosphate kinase	29.4	0.4	1.2	0.1	CY	CY	GO:0005737	NP
542	WP_000190880.1	LIC_11607	MRP family ATP-binding protein	260.9	NF	14.0	0.1	CY	CY	GO:0005737	NP
543	WP_001062346.1	LIC_11855	D-alanine--D-alanine ligase	36.5	NF	1.9	0.1	CY	CY	GO:0005737	P
544	WP_000054152.1	LIC_20238	hypothetical protein	953.9	45.8	5.0	0.1	CY	CY	GO:0005737	NP
545	WP_000576214.1	_	arylesterase	42.1	2.2	NF	0.1	PP	CY	GO:0005615	NP
546	WP_000062824.1	LIC_12859	MULTISPECIES: 30S ribosomal protein S8	1423.1	NF	74.8	0.1	CY	CY	GO:0005737	NP
547	WP_000150944.1	_	BolA family transcriptional regulator	65.5	3.4	NF	0.1	CY	CY	GO:0005737	NP
548	WP_000859505.1	LIC_12152	glucose-1-phosphate cytidylyltransferase	220.2	11.5	NF	0.1	CY	CY	GO:0005737	NP
549	WP_000391865.1	LIC_11556	MULTISPECIES: KH domain-containing protein	563.3	NF	29.4	0.1	CY	CY	GO:0005737	NP
550	WP_000566240.1	LIC_20064	3-oxoacyl-[acyl-carrier-protein] reductase	569.9	8.3	21.5	0.1	CY	CY	GO:0005737	NP
551	WP_000644947.1	LIC_12082	cysteine synthase A	6712.9	327.7	22.8	0.1	CY	CY	GO:0005737	NP
552	WP_000789400.1	LIC_12852	adenylate kinase	869.9	24.6	20.7	0.1	CY	CY	GO:0005737	NP
553	WP_001088908.1	LIC_11948	DUF4416 domain-containing protein	50.1	NF	2.6	0.1	UN	CY	GO:0005737	P
554	WP_000761500.1	LIC_10565	enoyl-CoA hydratase/isomerase family protein	920.3	39.5	8.3	0.1	CY	CY	GO:0005737	NP
555	WP_000659900.1	LIC_20100	HD family phosphohydrolase	6.0	0.3	NF	0.1	CY	CY	GO:0005737	NP
556	WP_000586163.1	LIC_11890	flagellin	3500.0	139.5	41.2	0.1	PP	CY	GO:0005737	P
557	WP_001165331.1	LIC_11423	RNA-binding protein	436.6	22.1	0.4	0.1	UN	EC	GO:0005737	NP
558	WP_001067557.1	LIC_12199	UDP-glucose 4-epimerase	677.3	2.4	32.4	0.1	CY	CY	GO:0005737	NP

559	WP_000590867.1	LIC_10948	ABC transporter ATP-binding protein	44.2	NF	2.3	0.1	CY	CY	GO:0005737	P
560	WP_000116243.1	LIC_11511	peptide deformylase	110.4	5.6	NF	0.1	CY	CY	GO:0005737	NP
561	WP_000091209.1	LIC_10756	30S ribosomal protein S7	2661.1	NF	136.0	0.1	CY	CY	GO:0005737	NP
562	WP_000102023.1	LIC_10272	elongation factor G	983.7	33.4	16.8	0.1	CY	CY	GO:0005737	NP
563	WP_000232823.1	LIC_11704	MULTISPECIES: 30S ribosomal protein S21	854.1	8.7	34.5	0.1	CY	CY	GO:0005737	NP
564	WP_001085747.1	LIC_11418	MULTISPECIES: ATP-dependent Clp protease ATP-binding subunit ClpX	69.7	NF	3.5	0.1	CY	CY	GO:0005737	P
565	WP_000899963.1	LIC_20223	hypothetical protein	9.6	NF	0.5	0.1	CY	CY	GO:0005737	NP
566	WP_002086035.1	_	FAD-binding oxidoreductase	161.7	1.3	6.8	0.1	CY	CY	GO:0005737	NP
567	WP_000536871.1	LIC_10748	MULTISPECIES: transcription termination/antitermination factor NusG	486.9	NF	24.4	0.1	CY	CY	GO:0005737	NP
568	WP_001217817.1	LIC_11653	hypothetical protein	106.3	5.3	NF	0.0	UN	PP	GO:0005615	P
569	WP_001104074.1	LIC_12515	ABC transporter substrate-binding protein	60.9	2.4	0.7	0.0	PP	OM	GO:0005737	P
570	WP_000016926.1	LIC_10358	hypothetical protein	26.4	0.6	0.7	0.0	CY	CY	GO:0005737	NP
571	WP_000078168.1	_	crotonyl-CoA carboxylase/reductase	1416.5	69.0	0.8	0.0	CY	PP	GO:0005737	NP
572	WP_000422649.1	LIC_12091	phosphoglycerate kinase	512.6	0.9	24.0	0.0	CY	CY	GO:0005737	NP
573	WP_001029963.1	LIC_11335	molecular chaperone GroEL	12569.5	256.8	354.8	0.0	CY	CY	GO:0005737	NP
574	WP_000805357.1	LIC_10428	UDP-N-acetylglucosamine diphosphorylase	89.3	NF	4.3	0.0	CY	CY	GO:0005737	NP
575	WP_000897116.1	_	hypothetical protein	21.8	1.1	NF	0.0	UN	OM	GO:0005737	NP

576	WP_001045300.1	_	dTDP-4-dehydrorhamnose 3,5-epimerase	144.3	2.3	4.7	0.0	UN	CY	GO:0005737	NP
577	WP_000594730.1	LIC_13023	hypothetical protein	37.4	NF	1.8	0.0	CY	CY	GO:0005886	NP
578	WP_000689546.1	LIC_12560	dUTP diphosphatase	151.0	7.3	NF	0.0	UN	CY	GO:0005737	NP
579	WP_000842289.1	_	uracil-DNA glycosylase	18.7	0.9	NF	0.0	CY	CY	GO:0005737	NP
580	WP_001188789.1	LIC_10750	50S ribosomal protein L1	715.1	2.4	31.6	0.0	CY	PP	GO:0005737	NP
581	WP_000002712.1	_	TIGR02300 family protein	336.3	NF	15.8	0.0	UN	CY	GO:0005737	NP
582	WP_001022053.1	LIC_10612	acetyltransferase	67.7	NF	3.2	0.0	CY	CY	GO:0005737	NP
583	WP_000614725.1	LIC_11655	bifunctional phosphoribosylaminoimidazolecarboxamide formyltransferase/inosine monophosphate cyclohydrolase	172.5	0.8	7.3	0.0	CY	CY	GO:0005737	NP
584	WP_001257925.1	LIC_13251	UDP-N-acetylglucosamine 1-carboxyvinyltransferase	262.4	0.4	11.9	0.0	CY	CY	GO:0005737	NP
585	WP_001015422.1	LIC_12188	class I SAM-dependent methyltransferase	739.4	9.7	24.6	0.0	CY	CY	GO:0005737	NP
586	WP_000291460.1	_	glycosyl hydrolase	17.4	0.4	0.4	0.0	CY	CY	GO:0005615	NP
587	WP_000361891.1	LIC_12810	hypothetical protein	21.7	NF	1.0	0.0	UN	PP	GO:0005886	NP
588	WP_000140212.1	LIC_10522	gfo/Idh/MocA family oxidoreductase	316.8	0.2	14.3	0.0	CY	CY	GO:0005737	NP
589	WP_001080218.1	_	glycine cleavage system protein T	136.1	2.4	3.8	0.0	CY	OM	GO:0005737	NP
590	WP_000196651.1	LIC_12545	MULTISPECIES: outer membrane lipoprotein carrier protein LolA	273.3	8.9	3.5	0.0	UN	OM	GO:0005886	P
591	WP_000090769.1	LIC_12849	MULTISPECIES: 30S ribosomal protein S13	1309.5	0.6	58.6	0.0	CY	CY	GO:0005737	NP

592	WP_000479982.1	LIC_12193	MULTISPECIES: hypothetical protein	199.3	1.4	7.6	0.0	CY	OM	GO:0005737	P
593	WP_000354445.1	LIC_11698	tyrosine--tRNA ligase	41.8	NF	1.9	0.0	CY	CY	GO:0005737	NP
594	WP_000098174.1	LIC_11847	ATP-binding protein	22.6	NF	1.0	0.0	IM	CY	GO:0005886	NP
595	WP_000389641.1	LIC_12705	translation initiation factor IF-2	650.2	21.5	7.7	0.0	CY	CY	GO:0005737	NP
596	WP_000998837.1	LIC_12694	glutamate synthase large subunit	691.4	25.8	5.0	0.0	CY	CY	GO:0005737	NP
597	WP_000033739.1	_	redox protein OsmC	123.9	5.5	NF	0.0	CY	CY	GO:0005737	NP
598	WP_000767694.1	LIC_10440	MULTISPECIES: nitrogen regulatory protein P-II	190.0	8.4	NF	0.0	IM	CY	GO:0005737	NP
599	WP_001970545.1	_	polyprenyl synthetase family protein	18.3	NF	0.8	0.0	CY	CY	GO:0005737	NP
600	WP_001076468.1	LIC_13345	glutamate--tRNA ligase	75.8	2.6	0.8	0.0	CY	CY	GO:0005737	NP
601	WP_001276053.1	_	DUF3383 domain-containing protein	58.8	NF	2.6	0.0	UN	OM	GO:0005737	P
602	WP_000855526.1	LIC_11822	3-isopropylmalate dehydratase large subunit	642.7	NF	28.3	0.0	CY	CY	GO:0005737	NP
603	WP_000852462.1	LIC_12829	MULTISPECIES: citrate synthase	732.6	14.2	17.9	0.0	CY	CY	GO:0005737	NP
604	WP_002146125.1	_	phosphoribosylformylglycinamidine cyclo-ligase	101.5	4.4	NF	0.0	CY	CY	GO:0005737	NP
605	WP_000346036.1	LIC_12168	DegT/DnrJ/EryC1/StrS family aminotransferase	282.4	11.7	0.6	0.0	CY	CY	GO:0005737	NP
606	WP_001110981.1	LIC_12877	hypothetical protein	13.3	NF	0.6	0.0	CY	CY	GO:0005737	P
607	WP_000180222.1	LIC_12211	heat-shock protein	1193.3	31.7	19.7	0.0	CY	CY	GO:0005737	P
608	WP_000714033.1	_	MULTISPECIES: lipoprotein LipL45	1362.1	45.8	12.9	0.0	CY	CY	GO:0005615	NP

609	WP_000392362.1	LIC_10521	YqgE/AlgH family protein	99.0	2.6	1.6	0.0	UN	OM	GO:0005737	NP
610	WP_000169688.1	LIC_11597	2-isopropylmalate synthase	48.6	0.5	1.6	0.0	CY	CY	GO:0005737	NP
611	WP_000565746.1	LIC_12233	MULTISPECIES: fructose-bisphosphate aldolase	2457.1	70.3	35.3	0.0	UN	CY	GO:0005737	NP
612	WP_000919338.1	LIC_11155	hypothetical protein	16.7	0.7	NF	0.0	CY	CY	GO:0005886	NP
613	WP_000135260.1	LIC_12847	30S ribosomal protein S4	1170.3	NF	49.7	0.0	CY	CY	GO:0005737	NP
614	WP_000907541.1	_	MULTISPECIES: hypothetical protein	5504.8	114.4	119.0	0.0	UN	CY	GO:0005737	NP
615	WP_000851065.1	LIC_11096	hypothetical protein	360.9	15.3	NF	0.0	UN	CY	GO:0005737	NP
616	WP_001237888.1	LIC_13153	UDP-glucose 4-epimerase GalE	167.5	NF	7.0	0.0	CY	CY	GO:0005737	NP
617	WP_000742439.1	LIC_12078	HD family phosphohydrolase	27.3	1.1	NF	0.0	CY	CY	GO:0005737	NP
618	WP_001053462.1	_	pirin family protein	161.0	NF	6.7	0.0	CY	CY	GO:0005737	NP
619	WP_000569939.1	LIC_12888	hypothetical protein	71.5	NF	3.0	0.0	UN	CY	GO:0005737	P
620	WP_000940600.1	LIC_12774	MULTISPECIES: 50S ribosomal protein L27	281.1	NF	11.6	0.0	CY	CY	GO:0005737	NP
621	WP_000447930.1	LIC_11920	DUF1577 domain-containing protein	130.6	NF	5.4	0.0	CY	CY	GO:0005737	NP
622	WP_001084828.1	LIC_10860	tryptophan synthase subunit alpha	63.3	1.6	1.0	0.0	CY	CY	GO:0005737	NP
623	WP_000028195.1	_	isocitrate lyase/phosphoenolpyruvate mutase family protein	103.8	0.6	3.7	0.0	CY	CY	GO:0005737	NP
624	WP_000656510.1	_	hypothetical protein	28.5	0.3	0.8	0.0	CY	OM	GO:0005737	P
625	WP_000671644.1	LIC_12923	spiro-SPASM protein	57.0	NF	2.3	0.0	CY	OM	GO:0005737	NP

626	WP_000951509.1	LIC_10386	MULTISPECIES: sigma-70 family RNA polymerase sigma factor	52.0	NF	2.1	0.0	CY	CY	GO:0005737	NP
627	WP_000086547.1	LIC_12858	MULTISPECIES: 50S ribosomal protein L6	1162.8	0.4	46.9	0.0	CY	CY	GO:0005737	NP
628	WP_000910893.1	LIC_11977	cAMP/cGMP-dependent 3',5'-cyclic-AMP/GMP phosphodiesterase	514.5	18.9	2.0	0.0	CY	CY	GO:0005886	NP
629	WP_000360599.1	LIC_13180	RNA pyrophosphohydrolase	64.5	2.6	NF	0.0	CY	CY	GO:0005737	NP
630	WP_000455556.1	LIC_10270	hydrolase	51.3	NF	2.1	0.0	CY	CY	GO:0005737	NP
631	WP_000049027.1	LIC_10670	DUF4256 domain-containing protein	67.0	0.7	2.0	0.0	CY	CY	GO:0005737	NP
632	WP_000932791.1	LIC_10023	MULTISPECIES: flagellar motor switch protein FliG	26.1	NF	1.0	0.0	CY	CY	GO:0005737	P
633	WP_000586170.1	LIC_11889	MULTISPECIES: flagellin	391.5	8.5	7.0	0.0	PP	PP	GO:0005737	P
634	WP_001071214.1	LIC_10835	hypothetical protein	37.2	NF	1.5	0.0	CY	CY	GO:0005737	NP
635	WP_000502718.1	LIC_11101	dihydroxy-acid dehydratase	518.8	20.3	NF	0.0	CY	CY	GO:0005737	NP
636	WP_001015340.1	LIC_10673	hypothetical protein	10.3	NF	0.4	0.0	CY	CY	GO:0005886	NP
637	WP_000212325.1	LIC_11656	phosphoribosylglycinamide formyltransferase	38.3	0.7	0.8	0.0	CY	CY	GO:0005737	NP
638	WP_001021982.1	LIC_10814	cytochrome c biogenesis protein	32.0	1.2	NF	0.0	UN	CY	GO:0005886	NP
639	WP_000658256.1	LIC_11213	hypothetical protein	209.4	8.1	NF	0.0	UN	EC	GO:0005737	P
640	WP_001089345.1	LIC_10309	glycine dehydrogenase	212.7	7.4	0.8	0.0	CY	CY	GO:0005886	NP
641	WP_000390637.1	LIC_10396	acetyl-CoA C-acetyltransferase	707.5	NF	27.4	0.0	CY	CY	GO:0005737	NP
642	WP_000993325.1	_	activator of HSP90 ATPase	105.7	4.1	NF	0.0	CY	CY	GO:0005737	NP

643	WP_000709511.1	LIC_13168	GDP-mannose 4,6-dehydratase	184.6	1.7	5.5	0.0	CY	CY	GO:0005737	NP
644	WP_000741297.1	LIC_12861	50S ribosomal protein L5	987.6	NF	38.0	0.0	CY	CY	GO:0005737	NP
645	WP_000457916.1	LIC_11980	twin-arginine translocase TatA/TatE family subunit	168.2	6.4	NF	0.0	IM	PP	GO:0005886	P
646	WP_000091770.1	LIC_12407	MULTISPECIES: type I glutamate--ammonia ligase	1733.7	32.2	33.8	0.0	CY	PP	GO:0005737	NP
647	WP_000064092.1	LIC_10789	transcription elongation factor GreA	457.7	8.8	8.6	0.0	CY	CY	GO:0005737	NP
648	WP_000708411.1	LIC_13311	UDP-N-acetylmuramate--alanine ligase	55.4	2.1	NF	0.0	CY	CY	GO:0005615	NP
649	WP_001199925.1	LIC_10382	acyl-CoA dehydrogenase	165.5	0.5	5.8	0.0	CY	OM	GO:0005737	NP
650	WP_000778965.1	LIC_12795	MULTISPECIES: acetyl-CoA C-acyltransferase	1567.7	6.0	53.2	0.0	CY	CY	GO:0005737	NP
651	WP_000636960.1	LIC_12668	DUF1554 domain-containing protein	11.3	0.4	NF	0.0	UN	EC	GO:0005886	P
652	WP_001972581.1	_	thioesterase	171.5	0.9	5.5	0.0	CY	CY	GO:0005737	NP
653	WP_000567189.1	LIC_12857	MULTISPECIES: 50S ribosomal protein L18	630.5	NF	23.6	0.0	CY	CY	GO:0005737	NP
654	WP_000868428.1	LIC_12850	MULTISPECIES: 50S ribosomal protein L36	85.4	NF	3.2	0.0	CY	CY		NP
655	WP_000687652.1	LIC_12474	MULTISPECIES: 2-oxoglutarate dehydrogenase subunit E1	498.7	3.9	14.7	0.0	CY	CY	GO:0005737	NP
656	WP_000950835.1	LIC_12866	MULTISPECIES: 50S ribosomal protein L16	1008.4	NF	37.5	0.0	CY	CY	GO:0005737	NP
657	WP_000388230.1	LIC_10591	divalent-cation tolerance protein CutA	56.1	2.1	NF	0.0	CY	CY	GO:0005737	P
658	WP_000157608.1	LIC_10074	diaminopimelate epimerase	178.7	3.2	3.4	0.0	CY	CY	GO:0005737	NP
659	WP_001253389.1	LIC_13361	DUF4139 domain-containing protein	72.6	2.6	0.0	0.0	UN	OM	GO:0005737	P

660	WP_000274334.1	LIC_10753	DNA-directed RNA polymerase subunit beta	1033.9	7.6	30.4	0.0	CY	CY	GO:0005737	NP
661	WP_000063746.1	LIC_10974	acetyl-CoA acetyltransferase	249.0	0.0	9.1	0.0	CY	CY	GO:0005737	NP
662	WP_001148872.1	LIC_10216	phosphoenolpyruvate carboxykinase (ATP)	1426.8	48.4	3.8	0.0	CY	CY	GO:0005737	NP
663	WP_000884017.1	LIC_12787	MULTISPECIES: anti-sigma factor antagonist	369.8	13.5	NF	0.0	CY	CY	GO:0005737	NP
664	WP_000727141.1	LIC_13413	phosphoribosylglycinamide formyltransferase 2	81.2	NF	2.9	0.0	IM	CY	GO:0005737	NP
665	WP_000136057.1	LIC_10631	MULTISPECIES: Fe-S cluster assembly ATPase SufC	14.2	NF	0.5	0.0	CY	CY	GO:0005737	NP
666	WP_001012068.1	LIC_12158	2,4-dihydroxyhept-2-ene-1,7-dioic acid aldolase	175.1	6.1	0.3	0.0	CY	CY	GO:0005737	NP
667	WP_001128536.1	LIC_13022	DUF1343 domain-containing protein	41.2	NF	1.5	0.0	CY	CY	GO:0005737	NP
668	WP_001202901.1	LIC_11366	hypothetical protein	1.6	0.1	NF	0.0	OM	OM	GO:0005886	P
669	WP_001972005.1	LIC_10788	flagellar filament outer layer protein Flaa	1068.8	13.4	25.2	0.0	CY	PP	GO:0005737	NP
670	WP_000939228.1	_	PhzF family phenazine biosynthesis protein	61.2	2.2	NF	0.0	CY	CY	GO:0005737	NP
671	WP_000374065.1	LIC_11976	acyl-CoA dehydrogenase	307.2	2.4	8.6	0.0	CY	CY	GO:0005737	NP
672	WP_002080787.1	LIC_12531	hypothetical protein	378.8	2.9	10.6	0.0	CY	CY	GO:0005737	NP
673	WP_000181768.1	LIC_12909	aspartate kinase	377.9	5.3	8.2	0.0	UN	CY	GO:0005737	NP
674	WP_000422624.1	LIC_20033	hypothetical protein	180.6	6.4	NF	0.0	CY	CY	GO:0005737	NP
675	WP_000684128.1	LIC_20022	NADPH-dependent oxidoreductase	105.5	NF	3.7	0.0	UN	CY	GO:0005886	NP
676	WP_000017465.1	LIC_10132	sigma-54-dependent Fis family transcriptional regulator	59.3	0.7	1.4	0.0	CY	CY	GO:0005737	NP

677	WP_000744700.1	LIC_10045	NAD-dependent epimerase/dehydratase family protein	384.7	6.6	7.0	0.0	CY	CY	GO:0005737	NP
678	WP_000734508.1	LIC_12895	hypothetical protein	144.5	NF	5.1	0.0	CY	CY	GO:0005615	NP
679	WP_000405900.1	LIC_12357	translational GTPase TypA	277.8	7.3	2.5	0.0	IM	CY	GO:0005737	NP
680	WP_001111376.1	_	FAD-dependent thymidylate synthase	127.6	3.5	1.0	0.0	CY	CY	GO:0005737	NP
681	WP_000736234.1	LIC_11883	hypothetical protein	11.2	0.4	NF	0.0	UN	OM	GO:0005886	P
682	WP_000769790.1	LIC_12499	hypothetical protein	20.7	0.7	NF	0.0	UN	OM	GO:0005615	P
683	WP_000837773.1	LIC_11605	hypothetical protein	69.1	2.4	NF	0.0	UN	CY	GO:0005737	NP
684	WP_000862891.1	LIC_10925	MULTISPECIES: hydrolase	84.6	NF	2.9	0.0	CY	CY	GO:0005737	NP
685	WP_000429410.1	LIC_11701	RNA polymerase sigma factor RpoD	571.8	3.9	15.7	0.0	CY	CY	GO:0005737	NP
686	WP_000438391.1	LIC_11220	Fe-S cluster assembly protein SufB	25.0	NF	0.9	0.0	CY	OM	GO:0005737	NP
687	WP_001288561.1	LIC_12925	citrate synthase	362.1	9.8	2.6	0.0	CY	CY	GO:0005737	NP
688	WP_000199612.1	LIC_10932	type I restriction-modification system subunit M	63.5	2.1	NF	0.0	CY	CY	GO:0005737	NP
689	WP_000838056.1	LIC_11327	flagellar P-ring protein	29.5	1.0	NF	0.0	PP	OM	GO:0005615	NP
690	WP_000016317.1	LIC_12695	hypothetical protein	64.0	0.9	1.2	0.0	CY	CY	GO:0005737	P
691	WP_000479709.1	_	VWA domain-containing protein	11.1	0.4	NF	0.0	UN	OM	GO:0005615	P
692	WP_000644954.1	LIC_13046	UDP-3-O-(3-hydroxymyristoyl)glucosamine N-acyltransferase	1003.5	0.2	33.1	0.0	CY	CY	GO:0005737	NP
693	WP_000532389.1	LIC_20016	hypothetical protein	716.4	3.7	20.0	0.0	IM	OM	GO:0005886	P

694	WP_000729197.1	_	MULTISPECIES: transcriptional regulator	113.0	1.9	1.8	0.0	CY	CY	GO:0005737	NP
695	WP_001137995.1	LIC_12883	carbamoyl phosphate synthase large subunit	198.6	0.1	6.4	0.0	UN	CY	GO:0005737	NP
696	WP_000202830.1	LIC_12166	alcohol dehydrogenase	471.5	3.4	12.0	0.0	CY	CY	GO:0005737	NP
697	WP_000509038.1	_	orotidine-5'-phosphate decarboxylase	90.7	NF	3.0	0.0	UN	OM	GO:0005737	NP
698	WP_001142358.1	LIC_10755	MULTISPECIES: 30S ribosomal protein S12	1601.5	NF	52.0	0.0	CY	CY	GO:0005737	NP
699	WP_000848056.1	_	enoyl-CoA hydratase	233.4	6.1	1.4	0.0	IM	CY	GO:0005737	NP
700	WP_000291591.1	LIC_11538	D-glycero-beta-D-manno-heptose-7-phosphate kinase	100.8	NF	3.2	0.0	CY	CY	GO:0005737	NP
701	WP_000586157.1	LIC_11531	flagellin	2006.9	30.2	34.1	0.0	PP	CY	GO:0005737	P
702	WP_001247305.1	LIC_10050	membrane protein	98.6	3.1	NF	0.0	OM	OM	GO:0005615	P
703	WP_001052498.1	LIC_13431	ABC transporter ATP-binding protein	45.4	NF	1.4	0.0	IM	CY	GO:0005737	P
704	WP_000067295.1	LIC_13142	phosphopantothenoylcysteine decarboxylase	156.6	NF	4.9	0.0	CY	CY	GO:0005737	NP
705	WP_001023796.1	LIC_10736	RidA family protein	165.3	5.2	NF	0.0	UN	PP	GO:0005737	NP
706	WP_001070122.1	LIC_12367	ribosome silencing factor	139.6	NF	4.4	0.0	CY	CY	GO:0005737	P
707	WP_001013415.1	LIC_12614	hypothetical protein	53.7	NF	1.7	0.0	UN	CY	GO:0005737	P
708	WP_000070933.1	_	activator of HSP90 ATPase	215.9	6.7	NF	0.0	CY	CY	GO:0005737	NP
709	WP_000170133.1	LIC_12765	2-Cys peroxiredoxin	507.0	NF	15.7	0.0	PP	PP	GO:0005737	P
710	WP_000483795.1	LIC_11361	M3 family peptidase	18.8	0.6	NF	0.0	CY	CY	GO:0005737	NP
711	WP_000951976.1	LIC_13318	ACP S-malonyltransferase	3575.5	98.2	12.4	0.0	UN	OM	GO:0005737	P

712	WP_000121286.1	LIC_13399	S-methyl-5'-thioadenosine phosphorylase	235.1	6.7	0.6	0.0	CY	CY	GO:0005737	NP
713	WP_000910559.1	LIC_10101	PLP-dependent transferase	30.2	NF	0.9	0.0	CY	CY	GO:0005737	NP
714	WP_000366622.1	LIC_11971	MULTISPECIES: hypothetical protein	230.9	3.6	3.5	0.0	CY	CY	GO:0005737	NP
715	WP_000451642.1	LIC_11758	acyl-CoA thioesterase	57.2	1.8	NF	0.0	CY	CY	GO:0005737	NP
716	WP_001227806.1	LIC_13001	DUF3604 domain-containing protein	31.4	1.0	NF	0.0	UN	PP	GO:0005886	P
717	WP_000013261.1	LIC_11604	hypothetical protein	175.7	NF	5.4	0.0	CY	CY	GO:0005737	NP
718	WP_000653653.1	_	short-chain dehydrogenase	57.9	NF	1.7	0.0	CY	CY	GO:0005737	NP
719	WP_000053472.1	LIC_11716	hypothetical protein	12.2	NF	0.4	0.0	CY	CY	GO:0005737	NP
720	WP_000262613.1	LIC_20085	methionine synthase	121.1	0.9	2.8	0.0	CY	CY	GO:0005737	NP
721	WP_000829480.1	LIC_12746	NADH-quinone oxidoreductase subunit F	99.4	0.1	2.9	0.0	CY	CY	GO:0005737	NP
722	WP_001291938.1	LIC_12958	MULTISPECIES: hypothetical protein	3307.9	19.5	79.5	0.0	UN	CY	GO:0005737	NP
723	WP_000358325.1	LIC_12543	30S ribosomal protein S12 methylthiotransferase RimO	98.4	NF	2.9	0.0	CY	CY	GO:0005737	NP
724	WP_000738272.1	LIC_11695	lipoprotein	143.3	3.1	1.1	0.0	UN	CY	GO:0005615	P
725	WP_000412102.1	LIC_12350	glycine--tRNA ligase	160.0	0.1	4.7	0.0	CY	CY	GO:0005737	NP
726	WP_000092003.1	LIC_20266	aspartate-semialdehyde dehydrogenase	190.5	5.6	NF	0.0	CY	CY	GO:0005737	NP
727	WP_000683574.1	LIC_12346	adenosine kinase	119.2	0.5	3.0	0.0	UN	CY	GO:0005737	NP
728	WP_000908611.1	LIC_10637	N-acetyltransferase	49.1	1.4	NF	0.0	UN	CY	GO:0005737	NP
729	WP_000439715.1	LIC_10571	homoserine dehydrogenase	99.3	NF	2.9	0.0	CY	CY	GO:0005737	NP

730	WP_001287342.1	LIC_13367	NADPH-dependent assimilatory sulfite reductase hemoprotein subunit	539.8	14.7	0.9	0.0	CY	CY	GO:0005737	NP
731	WP_001169645.1	LIC_12780	VOC family protein	69.3	2.0	NF	0.0	CY	EC	GO:0005737	P
732	WP_001191980.1	LIC_10699	esterase	157.5	4.5	NF	0.0	UN	CY	GO:0005737	NP
733	WP_000682938.1	LIC_12734	hypothetical protein	12.0	NF	0.3	0.0	UN	CY	GO:0005886	NP
734	WP_001068672.1	_	hypothetical protein	37.5	NF	1.1	0.0	CY	CY	GO:0005737	NP
735	WP_001032431.1	LIC_11243	MULTISPECIES: ATP synthase subunit beta	1489.1	2.7	39.6	0.0	CY	CY	GO:0005737	NP
736	WP_001291080.1	LIC_12495	crotonase	830.5	18.6	4.9	0.0	CY	CY	GO:0005737	NP
737	WP_000615785.1	LIC_12534	bifunctional 3,4-dihydroxy-2-butanone-4-phosphate synthase/GTP cyclohydrolase II	250.4	2.5	4.5	0.0	CY	CY	GO:0005737	NP
738	WP_000070955.1	LIC_11919	MULTISPECIES: IMP dehydrogenase	369.9	6.3	4.1	0.0	CY	CY	GO:0005737	NP
739	WP_000045686.1	LIC_10719	polyphosphate kinase 1	113.7	3.1	NF	0.0	IM	CY	GO:0005737	NP
740	WP_000691324.1	LIC_10350	phosphohistidine phosphatase SixA	79.6	2.2	NF	0.0	UN	CY	GO:0005737	NP
741	WP_001207830.1	_	DUF4254 domain-containing protein	142.7	3.9	NF	0.0	CY	CY	GO:0005737	P
742	WP_001083720.1	LIC_20001	carbohydrate-binding protein	256.9	7.0	0.1	0.0	OM	OM	GO:0005737	P
743	WP_001257938.1	_	hypothetical protein	131.3	0.7	2.9	0.0	UN	OM	GO:0005737	P
744	WP_000007881.1	_	class 1 fructose-bisphosphatase	89.8	2.5	NF	0.0	CY	OM	GO:0005737	NP
745	WP_000167663.1	LIC_11364	NAD(P)-dependent oxidoreductase	145.7	0.6	3.4	0.0	CY	CY	GO:0005886	NP
746	WP_000290471.1	LIC_12444	MULTISPECIES: 50S ribosomal protein L32	1010.5	NF	27.4	0.0	CY	CY	GO:0005737	NP
747	WP_001275641.1	LIC_11244	ATP synthase epsilon chain	302.1	NF	8.2	0.0	UN	CY	GO:0005737	NP

748	WP_000365849.1	_	cell division protein ZapA	128.8	3.5	NF	0.0	CY	CY	GO:0005737	NP
749	WP_001281293.1	_	prephenate dehydratase	53.7	1.5	NF	0.0	CY	CY	GO:0005737	NP
750	WP_001159758.1	LIC_12126	dTDP-4-dehydrorhamnose 3,5-epimerase	22.2	NF	0.6	0.0	CY	CY	GO:0005737	NP
751	WP_000116258.1	_	2-isopropylmalate synthase	175.4	1.4	3.3	0.0	CY	CY	GO:0005737	NP
752	WP_000865278.1	LIC_12248	pyridoxine 5'-phosphate synthase	38.0	1.0	NF	0.0	CY	CY	GO:0005737	NP
753	WP_000836885.1	_	hypothetical protein	7.5	0.2	NF	0.0	CY	OM	GO:0005886	P
754	WP_000956231.1	LIC_20101	MULTISPECIES: chemoreceptor glutamine deamidase CheD	32.0	NF	0.9	0.0	UN	CY	GO:0005737	NP
755	WP_001199946.1	LIC_10517	leucine--tRNA ligase	51.2	1.4	NF	0.0	CY	CY	GO:0005737	NP
756	WP_000789319.1	LIC_13163	3-methyl-2-oxobutanoate hydroxymethyltransferase	69.9	1.9	NF	0.0	CY	CY	GO:0005737	NP
757	WP_001141641.1	LIC_11438	peptidylprolyl isomerase	343.6	3.2	5.9	0.0	CY	CY	GO:0005737	NP
758	WP_001141234.1	LIC_10140	chorismate synthase	149.1	3.9	NF	0.0	CY	CY	GO:0005737	NP
759	WP_000504720.1	LIC_11745	DNA recombination/repair protein RecA	339.8	0.5	8.3	0.0	CY	CY	GO:0005737	NP
760	WP_000141824.1	LIC_13231	formylglycine-generating enzyme family protein	30.7	0.8	NF	0.0	UN	OM	GO:0005615	P
761	WP_000762682.1	LIC_10733	cytosol aminopeptidase	248.1	6.2	0.1	0.0	CY	CY	GO:0005737	NP
762	WP_001019283.1	LIC_11636	TIGR00266 family protein	93.0	2.4	NF	0.0	UN	EC	GO:0005737	P
763	WP_000391049.1	LIC_10287	6-carboxytetrahydropterin synthase QueD	110.1	2.8	NF	0.0	CY	CY	GO:0005737	NP
764	WP_000690652.1	LIC_11552	methionyl-tRNA formyltransferase	22.2	0.6	NF	0.0	CY	CY	GO:0005737	NP

765	WP_000608295.1	LIC_12197	DegT/DnrJ/EryC1/StrS family aminotransferase	422.9	10.5	0.3	0.0	CY	CY	GO:0005737	NP
766	WP_000042798.1	LIC_11791	helicase	320.8	1.0	7.2	0.0	CY	CY	GO:0005737	NP
767	WP_000023116.1	_	aldo/keto reductase	215.4	5.5	NF	0.0	CY	CY	GO:0005737	NP
768	WP_000671159.1	LIC_11558	tRNA (guanosine(37)-N1)-methyltransferase TrmD	11.6	0.3	NF	0.0	CY	CY	GO:0005737	NP
769	WP_000181227.1	LIC_13258	ferredoxin	32.4	0.8	NF	0.0	CY	CY	GO:0005737	NP
770	WP_000127192.1	LIC_11273	DUF2225 domain-containing protein	183.1	1.5	3.2	0.0	CY	CY	GO:0005737	NP
771	WP_001083687.1	_	putative peptidyl-prolyl cis-trans isomerase	31.9	0.8	NF	0.0	UN	CY	GO:0005737	NP
772	WP_000799620.1	LIC_10664	ATPase	626.6	15.8	NF	0.0	UN	CY	GO:0005737	NP
773	WP_000636852.1	LIC_12319	serine/threonine-protein phosphatase	32.5	NF	0.8	0.0	CY	OM	GO:0005737	NP
774	WP_000433586.1	LIC_10451	MULTISPECIES: hypothetical protein	78.0	2.0	NF	0.0	CY	CY	GO:0005737	NP
775	WP_000752686.1	LIC_12848	MULTISPECIES: 30S ribosomal protein S11	1107.8	NF	27.9	0.0	CY	CY	GO:0005737	NP
776	WP_000918546.1	LIC_10269	cAMP-binding protein	51.4	NF	1.3	0.0	CY	CY	GO:0005737	NP
777	WP_000577498.1	_	YceI family protein	39.7	1.0	NF	0.0	CY	CY	GO:0005886	NP
778	WP_001158527.1	_	AraC family transcriptional regulator	1.2	0.0	NF	0.0	IM	IM	GO:0005886	P
779	WP_001126680.1	LIC_12257	N-acetyltransferase	13.3	0.3	NF	0.0	CY	CY	GO:0005737	NP
780	WP_000334327.1	LIC_10445	MULTISPECIES: glutamine--fructose-6-phosphate aminotransferase	221.3	0.5	5.0	0.0	CY	CY	GO:0005737	NP
781	WP_000889241.1	LIC_10339	ATP-dependent Clp protease ATP-binding subunit	289.7	5.2	2.0	0.0	CY	CY	GO:0005737	NP

782	WP_000822886.1	LIC_11357	alcohol dehydrogenase	99.5	2.5	NF	0.0	CY	CY	GO:0005737	NP
783	WP_000235575.1	LIC_20115	sigma-70 family RNA polymerase sigma factor	111.3	NF	2.8	0.0	CY	CY	GO:0005737	NP
784	WP_000757856.1	_	methylenetetrahydrofolate reductase [NAD(P)H]	22.4	NF	0.5	0.0	CY	CY	GO:0005737	NP
785	WP_001202091.1	LIC_10363	isovaleryl-CoA dehydrogenase	290.2	NF	7.1	0.0	CY	CY	GO:0005737	NP
786	WP_000681221.1	LIC_13325	MULTISPECIES: phosphopantetheine adenylyltransferase	64.6	NF	1.6	0.0	CY	CY	GO:0005737	NP
787	WP_000051773.1	LIC_11182	hypothetical protein	2152.7	44.5	7.8	0.0	PP	PP	GO:0005737	P
788	WP_001227585.1	LIC_10787	MULTISPECIES: endoflagellar filament sheath protein	1392.5	12.7	20.6	0.0	CY	CY	GO:0005615	NP
789	WP_000582222.1	LIC_12187	DegT/DnrJ/EryC1/StrS family aminotransferase	159.6	0.9	2.9	0.0	CY	CY	GO:0005737	NP
790	WP_000013204.1	LIC_10751	50S ribosomal protein L10	874.2	1.7	19.0	0.0	CY	CY	GO:0005737	NP
791	WP_000795784.1	_	hypothetical protein	2326.9	51.6	3.5	0.0	EC	EC	GO:0005886	P
792	WP_000620380.1	LIC_12952	PEGA domain-containing protein	8.7	0.2	NF	0.0	IM	OM	GO:0005886	P
793	WP_001166720.1	LIC_13263	ribonuclease D	76.2	0.9	0.9	0.0	CY	CY	GO:0005737	NP
794	WP_001062719.1	LIC_13244	MULTISPECIES: isocitrate dehydrogenase (NADP(+))	2169.0	42.6	8.6	0.0	CY	CY	GO:0005737	NP
795	WP_000004301.1	LIC_10710	hypothetical protein	149.8	0.6	2.9	0.0	CY	CY	GO:0005737	NP
796	WP_000698533.1	_	pyruvate kinase	82.6	1.0	1.0	0.0	CY	CY	GO:0005737	NP

797	WP_000729204.1	LIC_10749	MULTISPECIES: 50S ribosomal protein L11	916.4	5.5	15.8	0.0	CY	PP	GO:0005737	NP
798	WP_000677991.1	_	bacterioferritin	1554.4	19.0	17.0	0.0	CY	CY	GO:0005886	NP
799	WP_000712694.1	LIC_12828	transglycosylase	12.2	0.1	0.2	0.0	UN	CY	GO:0005615	P
800	WP_001084413.1	LIC_11163	MULTISPECIES: DUF1963 domain-containing protein	33.3	NF	0.8	0.0	CY	CY	GO:0005737	NP
801	WP_000672100.1	LIC_11622	MULTISPECIES: biopolymer transporter ExbD	264.9	NF	6.0	0.0	UN	CY	GO:0005886	NP
802	WP_000365024.1	LIC_12551	acyl-CoA dehydrogenase	287.9	0.3	6.1	0.0	CY	CY	GO:0005886	NP
803	WP_001127234.1	LIC_10444	glucose-1-phosphate thymidylyltransferase	64.4	1.4	NF	0.0	CY	CY	GO:0005737	NP
804	WP_000394249.1	_	MULTISPECIES: F0F1 ATP synthase subunit C	2385.2	NF	52.9	0.0	UN	PP	GO:0005886	NP
805	WP_000511542.1	LIC_12870	50S ribosomal protein L2	2057.8	0.3	45.3	0.0	CY	EC	GO:0005737	NP
806	WP_001076098.1	LIC_10039	type I 3-dehydroquinate dehydratase	13.9	NF	0.3	0.0	CY	CY	GO:0005737	NP
807	WP_000884059.1	LIC_12203	MULTISPECIES: phosphoheptose isomerase	129.1	0.7	2.1	0.0	CY	CY	GO:0005737	NP
808	WP_000611594.1	LIC_12189	cupin fold metalloprotein, WbuC family	148.1	NF	3.3	0.0	CY	CY	GO:0005737	NP
809	WP_000477913.1	LIC_20212	hypothetical protein	73.9	1.6	NF	0.0	UN	OM	GO:0005886	P
810	WP_000551848.1	LIC_10032	molecular chaperone Hsp33	55.1	NF	1.2	0.0	CY	CY	GO:0005737	NP
811	WP_001071499.1	LIC_10969	CoA transferase	193.8	4.2	NF	0.0	CY	CY	GO:0005737	P
812	WP_001256473.1	LIC_10754	DNA-directed RNA polymerase subunit beta'	1096.6	6.1	17.7	0.0	CY	CY	GO:0005737	NP
813	WP_011172333.1	_	ribosome assembly cofactor RimP	69.6	NF	1.5	0.0	CY	CY	GO:0005737	NP
814	WP_001966744.1	LIC_10762	30S ribosomal protein S9	958.5	NF	20.8	0.0	CY	CY	GO:0005737	NP

815	WP_000076460.1	LIC_10005	DNA topoisomerase (ATP-hydrolyzing) subunit B	187.7	3.8	0.2	0.0	CY	CY	GO:0005737	NP
816	WP_001111751.1	LIC_12565	adenylosuccinate synthetase	477.7	10.3	NF	0.0	CY	CY	GO:0005737	NP
817	WP_000079225.1	LIC_10833	VOC family protein	51.4	NF	1.1	0.0	UN	PP	GO:0005737	P
818	WP_000742261.1	LIC_11456	lipoprotein LipL31	1099.2	4.8	18.8	0.0	UN	CY	GO:0005615	NP
819	WP_000716064.1	LIC_10690	WGR domain-containing protein	228.9	NF	4.9	0.0	CY	CY	GO:0005737	P
820	WP_000501205.1	LIC_11355	MULTISPECIES: transketolase	492.4	NF	10.5	0.0	CY	CY	GO:0005737	NP
821	WP_002072562.1	LIC_10973	porin OmpL1	1987.8	8.1	33.7	0.0	UN	EC	GO:0005615	P
822	WP_001002770.1	LIC_11461	Asp-tRNA(Asn)/Glu-tRNA(Gln) amidotransferase GatCAB subunit A	273.4	0.4	5.3	0.0	CY	OM	GO:0005737	NP
823	WP_000734456.1	LIC_12139	UDP-N-acetylglucosamine 2-epimerase (non-hydrolyzing)	67.2	1.2	0.2	0.0	CY	CY	GO:0005737	NP
824	WP_000802848.1	LIC_10235	MULTISPECIES: hypothetical protein	537.6	2.7	8.5	0.0	UN	CY	GO:0005737	P
825	WP_000890370.1	LIC_11529	4-hydroxy-3-methylbut-2-enyl diphosphate reductase	52.6	1.1	NF	0.0	CY	CY	GO:0005737	NP
826	WP_000587238.1	_	MULTISPECIES: acyl-CoA dehydrogenase	587.4	0.3	11.9	0.0	CY	CY	GO:0005886	NP
827	WP_000082216.1	LIC_10843	4-hydroxy-tetrahydrodipicolinate reductase	186.8	1.7	2.1	0.0	CY	CY	GO:0005737	NP
828	WP_000875567.1	LIC_10368	hypothetical protein	245.3	5.0	NF	0.0	UN	EC	GO:0005737	P
829	WP_001113793.1	LIC_10076	MULTISPECIES: hypothetical protein	79.5	1.6	NF	0.0	UN	CY	GO:0005737	P
830	WP_000086813.1	_	bifunctional porphobilinogen deaminase/uroporphyrinogen synthase	60.4	1.2	NF	0.0	CY	OM	GO:0005737	NP

831	WP_000837582.1	LIC_13392	hypothetical protein	809.6	0.6	15.7	0.0	UN	PP	GO:0005615	P
832	WP_000842773.1	LIC_12030	alpha/beta hydrolase	69.9	1.4	NF	0.0	UN	OM	GO:0005737	NP
833	WP_000440816.1	_	signal peptide peptidase SppA	558.3	NF	11.1	0.0	IM	OM	GO:0005886	NP
834	WP_001972951.1	LIC_13347	DNA gyrase subunit A	313.0	5.9	0.2	0.0	CY	CY	GO:0005737	NP
835	WP_000494006.1	LIC_12535	riboflavin synthase	73.6	1.4	NF	0.0	CY	CY	GO:0005737	NP
836	WP_000895907.1	_	DUF1993 domain-containing protein	159.5	NF	3.1	0.0	CY	CY	GO:0005737	NP
837	WP_000428541.1	LIC_20058	MULTISPECIES: methylmalonyl-CoA mutase	1010.4	9.2	10.5	0.0	CY	CY	GO:0005737	NP
838	WP_001226991.1	LIC_10834	dihydrofolate reductase	40.1	NF	0.8	0.0	UN	CY	GO:0005737	NP
839	WP_002153498.1	LIC_12860	MULTISPECIES: 30S ribosomal protein S14 type Z	4887.1	NF	95.0	0.0	CY	CY	GO:0005737	NP
840	WP_000284477.1	LIC_12465	threonine--tRNA ligase	358.7	0.7	6.2	0.0	CY	CY	GO:0005737	NP
841	WP_000202873.1	LIC_12689	ATP-dependent DNA helicase RecQ	11.1	0.2	NF	0.0	CY	CY	GO:0005737	NP
842	WP_000110035.1	LIC_12476	dihydrolipoyllysine-residue succinyltransferase	1132.2	15.1	6.6	0.0	CY	CY	GO:0005737	NP
843	WP_001007972.1	LIC_12027	acetylesterase	198.5	0.1	3.7	0.0	CY	CY	GO:0005737	NP
844	WP_000694669.1	_	hypothetical protein	32.0	NF	0.6	0.0	CY	CY	GO:0005737	NP
845	WP_000780086.1	LIC_11609	3-polyprenyl-4-hydroxybenzoate decarboxylase	42.8	0.8	NF	0.0	CY	CY	GO:0005737	NP
846	WP_000695903.1	LIC_11025	MULTISPECIES: anti-sigma factor antagonist	150.5	NF	2.8	0.0	CY	CY	GO:0005737	NP
847	WP_000669336.1	LIC_13056	MULTISPECIES: endoflagellar motor protein	48.7	0.9	NF	0.0	IM	CY	GO:0005737	P

848	WP_000228909.1	_	thioredoxin domain-containing protein	15.6	NF	0.3	0.0	CY	CY	GO:0005737	NP
849	WP_000448574.1	LIC_12766	anti-sigma factor antagonist	164.2	3.1	NF	0.0	UN	CY	GO:0005737	NP
850	WP_001093510.1	LIC_12292	5-(carboxyamino)imidazole ribonucleotide mutase	115.2	0.9	1.2	0.0	UN	CY	GO:0005737	NP
851	WP_001982325.1	_	phosphoglycerate dehydrogenase	180.8	3.2	0.2	0.0	CY	CY	GO:0005737	NP
852	WP_000715958.1	LIC_12192	hypothetical protein	71.5	NF	1.3	0.0	CY	CY	GO:0005737	NP
853	WP_000638432.1	LIC_11778	adenosylmethionine--8-amino-7-oxononanoate transaminase	12.3	0.2	NF	0.0	CY	CY	GO:0005737	NP
854	WP_001183065.1	LIC_10460	VOC family protein	177.9	3.2	NF	0.0	UN	CY	GO:0005737	P
855	WP_000599558.1	_	hypothetical protein	148.8	0.7	2.0	0.0	UN	OM	GO:0005737	P
856	WP_001026144.1	_	MULTISPECIES: hypothetical protein	298.3	5.4	NF	0.0	CY	CY	GO:0005615	P
857	WP_000613920.1	LIC_10403	6,7-dimethyl-8-ribityllumazine synthase	313.7	4.7	0.9	0.0	CY	CY	GO:0005737	NP
858	WP_000570103.1	LIC_12470	hypothetical protein	28.5	NF	0.5	0.0	CY	CY	GO:0005737	NP
859	WP_000146501.1	LIC_11298	dehypoxanthine futalosine cyclase	81.4	0.3	1.1	0.0	CY	CY	GO:0005737	NP
860	WP_000825971.1	LIC_10586	DNA polymerase I	28.0	0.5	NF	0.0	CY	CY	GO:0005737	NP
861	WP_000342713.1	LIC_12195	transketolase	324.5	NF	5.8	0.0	CY	CY	GO:0005737	NP
862	WP_000647275.1	LIC_12872	50S ribosomal protein L4	2010.7	NF	35.8	0.0	CY	PP	GO:0005737	NP
863	WP_001104042.1	LIC_20259	MULTISPECIES: hypothetical protein	388.6	NF	6.9	0.0	CY	CY	GO:0005737	NP
864	WP_002074675.1	_	phosphoribosylamine--glycine ligase	67.4	1.2	NF	0.0	CY	PP	GO:0005737	NP
865	WP_000665953.1	LIC_12733	MULTISPECIES: methylglyoxal synthase	179.3	1.8	1.4	0.0	CY	CY	GO:0005737	NP

866	WP_000130165.1	LIC_10112	imidazoleglycerol-phosphate dehydratase	137.5	2.4	NF	0.0	CY	CY	GO:0005737	NP
867	WP_001288005.1	LIC_20044	molecular chaperone HtpG	1577.6	15.4	11.9	0.0	CY	CY	GO:0005737	NP
868	WP_000529920.1	LIC_11896	DNA-binding response regulator	22.7	0.4	NF	0.0	CY	CY	GO:0005737	NP
869	WP_000876121.1	LIC_12548	MULTISPECIES: hypothetical protein	206.8	3.6	NF	0.0	UN	CY	GO:0005737	NP
870	WP_000695329.1	LIC_11241	ATP synthase subunit alpha	2218.0	7.6	30.5	0.0	CY	CY	GO:0005737	NP
871	WP_000777483.1	LIC_13219	phenylalanine--tRNA ligase subunit beta	144.1	2.5	NF	0.0	CY	CY	GO:0005737	NP
872	WP_000767698.1	LIC_10431	MULTISPECIES: aminoacyl-tRNA hydrolase	207.3	NF	3.5	0.0	CY	CY	GO:0005737	NP
873	WP_000694733.1	LIC_10127	MBL fold metallo-hydrolase	167.4	2.8	NF	0.0	CY	CY	GO:0005737	P
874	WP_001024432.1	LIC_11460	Asp-tRNA(Asn)/Glu-tRNA(Gln) amidotransferase GatCAB subunit C	384.4	6.5	NF	0.0	CY	CY	GO:0005737	NP
875	WP_000202680.1	LIC_12536	bifunctional diaminohydroxyphosphoribosylaminopyrimidine deaminase/5-amino-6-(5-phosphoribosylamino)uracil reductase	63.9	1.1	NF	0.0	CY	CY	GO:0005737	NP
876	WP_000721426.1	LIC_12865	50S ribosomal protein L29	1763.2	8.7	20.8	0.0	UN	CY	GO:0005737	NP
877	WP_000866993.1	_	TetR/AcrR family transcriptional regulator	63.2	NF	1.1	0.0	CY	CY	GO:0005737	NP
878	WP_000453665.1	LIC_10415	hypothetical protein	56.7	0.9	NF	0.0	CY	CY	GO:0005737	P
879	WP_001095154.1	_	hypothetical protein	152.1	0.6	2.0	0.0	CY	CY	GO:0005737	NP
880	WP_000163348.1	LIC_11089	metallo-mystery pair system four-Cys motif protein	165.7	2.7	NF	0.0	UN	EC	GO:0005886	P
881	WP_001001876.1	LIC_12210	Hsp20/alpha crystallin family protein	1598.0	11.2	15.0	0.0	CY	CY	GO:0005737	P

882	WP_001238476.1	LIC_13184	murein transglycosylase	5.6	0.1	NF	0.0	UN	OM	GO:0005615	NP
883	WP_000675255.1	LIC_12153	dehydrogenase	97.5	NF	1.6	0.0	CY	CY	GO:0005737	NP
884	WP_001137844.1	LIC_11852	O-acetylhomoserine aminocarboxypropyltransferase/cysteine synthase	503.0	8.1	NF	0.0	CY	PP	GO:0005737	NP
885	WP_000957005.1	LIC_12460	hypothetical protein	391.0	3.3	3.0	0.0	CY	CY	GO:0005737	NP
886	WP_000825586.1	LIC_11274	hypothetical protein	422.7	2.3	4.5	0.0	CY	CY	GO:0005737	NP
887	WP_001220497.1	LIC_11800	MULTISPECIES: MBL fold metallo-hydrolase	74.5	0.3	0.9	0.0	CY	CY	GO:0005737	NP
888	WP_001053850.1	LIC_11647	rRNA pseudouridine synthase	17.1	0.1	0.1	0.0	CY	CY	GO:0005737	NP
889	WP_001062325.1	_	2-oxo acid dehydrogenase subunit E2	310.3	3.2	1.7	0.0	CY	OM	GO:0005737	NP
890	WP_001060166.1	LIC_10949	aldo/keto reductase	97.6	1.5	NF	0.0	CY	CY	GO:0005737	NP
891	WP_000450798.1	LIC_12421	ATPase	215.7	0.2	3.1	0.0	CY	CY	GO:0005886	NP
892	WP_001983897.1	_	glutathione peroxidase	111.9	NF	1.7	0.0	CY	CY	GO:0005737	P
893	WP_001096782.1	LIC_12862	50S ribosomal protein L24	1064.2	NF	16.6	0.0	CY	CY	GO:0005737	NP
894	WP_000453256.1	LIC_10470	peptide chain release factor 2	199.5	3.1	NF	0.0	CY	CY	GO:0005737	NP
895	WP_000535110.1	LIC_11381	MULTISPECIES: polymerase	60.4	0.8	0.2	0.0	CY	CY	GO:0005737	NP
896	WP_000807227.1	LIC_10221	Asp-tRNA(Asn)/Glu-tRNA(Gln) amidotransferase GatCAB subunit B	335.1	4.4	0.7	0.0	CY	CY	GO:0005737	NP
897	WP_000586161.1	_	MULTISPECIES: flagellin	156.7	2.4	NF	0.0	PP	EC	GO:0005737	P
898	WP_001012709.1	LIC_10429	ribose-phosphate pyrophosphokinase	217.2	NF	3.3	0.0	CY	CY	GO:0005737	NP

899	WP_001973732.1	LIC_11541	2-dehydro-3-deoxyphosphooctonate aldolase	135.5	1.5	0.6	0.0	CY	CY	GO:0005737	NP
900	WP_000344487.1	LIC_12186	methionyl-tRNA formyltransferase	142.4	2.1	NF	0.0	CY	CY	GO:0005737	NP
901	WP_000460443.1	_	ankyrin repeat domain-containing protein	13.6	NF	0.2	0.0	CY	CY	GO:0005737	NP
902	WP_000372296.1	LIC_10874	4Fe-4S dicluster domain-containing protein	1497.8	NF	22.0	0.0	IM	OM	GO:0005737	NP
903	WP_000723989.1	LIC_20185	iron dicitrate transporter FecR	4844.9	34.8	36.2	0.0	UN	OM	GO:0005886	NP
904	WP_001968786.1	_	hypothetical protein	61.4	0.9	NF	0.0	CY	CY	GO:0005737	NP
905	WP_001049451.1	LIC_12581	MULTISPECIES: ATPase	129.5	NF	1.9	0.0	UN	EC	GO:0005737	P
906	WP_000238902.1	LIC_10141	NADH dehydrogenase	153.6	0.4	1.8	0.0	CY	CY	GO:0005737	NP
907	WP_000980946.1	LIC_11897	pyruvate dehydrogenase (acetyl-transferring) E1 component subunit alpha	108.3	NF	1.6	0.0	CY	CY	GO:0005737	NP
908	WP_000276952.1	LIC_13280	dehydratase	60.6	0.9	NF	0.0	UN	CY	GO:0005737	NP
909	WP_001173879.1	LIC_11491	lipid A Kdo2 1-phosphate O-methyltransferase	44.9	0.3	0.3	0.0	IM	IM	GO:0005886	NP
910	WP_001178919.1	LIC_12523	DUF2236 domain-containing protein	84.6	NF	1.2	0.0	CY	CY	GO:0005737	NP
911	WP_000050899.1	LIC_10198	inositol monophosphatase	340.3	4.8	NF	0.0	CY	CY	GO:0005737	NP
912	WP_000179852.1	LIC_10852	MULTISPECIES: UMP kinase	238.0	3.3	NF	0.0	CY	CY	GO:0005737	NP
913	WP_000802930.1	LIC_10763	alanine--tRNA ligase	81.2	0.1	1.0	0.0	CY	OM	GO:0005737	NP
914	WP_000833301.1	_	sigma factor regulatory protein FecR	356.2	0.1	4.8	0.0	UN	EC	GO:0005615	NP
915	WP_000124812.1	LIC_10570	MULTISPECIES: anti-sigma factor antagonist	130.7	1.8	NF	0.0	CY	CY	GO:0005737	NP
916	WP_000841027.1	_	branched-chain amino acid transaminase	256.8	3.2	0.3	0.0	CY	OM	GO:0005737	NP

917	WP_000503780.1	LIC_11370	flagellar motor switch protein FliN	154.1	0.7	1.5	0.0	IM	OM	GO:0005737	P
918	WP_000450441.1	LIC_20108	anti-sigma factor antagonist	269.2	2.1	1.6	0.0	UN	CY	GO:0005737	NP
919	WP_000843310.1	LIC_11003	lipoprotein LipL71	4387.2	26.7	33.4	0.0	OM	OM	GO:0005886	P
920	WP_000799638.1	LIC_12181	WxcM-like domain-containing protein	43.6	NF	0.6	0.0	UN	CY	GO:0005737	NP
921	WP_000250249.1	_	MULTISPECIES: hypothetical protein	212.6	NF	2.9	0.0	UN	CY	GO:0005737	P
922	WP_000913341.1	_	orotate phosphoribosyltransferase	201.0	0.9	1.9	0.0	CY	CY	GO:0005737	NP
923	WP_000136083.1	LIC_11836	MULTISPECIES: flagellar motor switch protein FliM	60.3	NF	0.8	0.0	IM	CY	GO:0005737	P
924	WP_000861675.1	LIC_12294	UDP-glucose/GDP-mannose dehydrogenase family protein	208.0	0.8	1.9	0.0	UN	CY	GO:0005737	NP
925	WP_000801906.1	LIC_12772	glutamate 5-kinase	17.2	0.2	NF	0.0	CY	OM	GO:0005737	NP
926	WP_000111953.1	LIC_20226	methylthioribulose 1-phosphate dehydratase	99.2	NF	1.3	0.0	CY	CY	GO:0005737	NP
927	WP_001157779.1	_	insulinase family protein	21.0	0.3	NF	0.0	UN	CY	GO:0005737	NP
928	WP_000763025.1	LIC_12450	prephenate dehydrogenase/arogenate dehydrogenase family protein	25.1	NF	0.3	0.0	UN	OM	GO:0005615	NP
929	WP_001114726.1	LIC_11600	HslU--HslV peptidase proteolytic subunit	147.0	1.9	NF	0.0	CY	CY	GO:0005737	NP
930	WP_000808789.1	LIC_12589	NUDIX hydrolase	335.5	0.7	3.6	0.0	CY	CY	GO:0005737	NP
931	WP_001089002.1	LIC_13129	methionine--tRNA ligase	141.3	0.5	1.3	0.0	CY	CY	GO:0005737	NP
932	WP_000423512.1	_	nucleoside triphosphate pyrophosphohydrolase	22.6	NF	0.3	0.0	CY	CY	GO:0005737	NP

933	WP_001046757.1	LIC_12167	acylneuraminate cytidylyltransferase family protein	137.5	NF	1.7	0.0	CY	CY	GO:0005737	NP
934	WP_000805070.1	LIC_13088	MULTISPECIES: DNA-binding response regulator	206.7	2.4	0.2	0.0	CY	CY	GO:0005737	NP
935	WP_001022640.1	LIC_12155	inositol phosphatase	45.9	NF	0.6	0.0	UN	OM	GO:0005737	NP
936	WP_000595826.1	LIC_20158	ATP-dependent protease	17.3	0.2	NF	0.0	CY	CY	GO:0005737	NP
937	WP_000409183.1	LIC_20078	MULTISPECIES: hypothetical protein	1308.4	0.2	16.0	0.0	CY	CY	GO:0005737	NP
938	WP_000853488.1	LIC_12964	quinolinate synthase	61.3	NF	0.8	0.0	CY	CY	GO:0005737	NP
939	WP_001075132.1	LIC_20011	glutamate-1-semialdehyde 2,1-aminomutase	125.7	1.6	NF	0.0	CY	CY	GO:0005737	NP
940	WP_000808030.1	LIC_11746	N-acetyl-gamma-glutamyl-phosphate reductase	84.9	1.0	NF	0.0	CY	CY	GO:0005737	NP
941	WP_001123874.1	_	bifunctional glutamate N-acetyltransferase/amino-acid acetyltransferase ArgJ	19.8	NF	0.2	0.0	CY	CY	GO:0005737	NP
942	WP_000529953.1	LIC_12867	30S ribosomal protein S3	1109.1	0.8	12.8	0.0	CY	CY	GO:0005737	NP
943	WP_000365065.1	LIC_13370	sulfate adenylyltransferase	178.7	0.9	1.3	0.0	CY	CY	GO:0005737	NP
944	WP_002075981.1	_	oxidoreductase	14.9	NF	0.2	0.0	UN	CY	GO:0005737	NP
945	WP_000538229.1	LIC_13471	transcriptional regulator	383.9	4.6	NF	0.0	CY	CY	GO:0005737	NP
946	WP_000600889.1	LIC_12176	NAD-dependent dehydratase	43.4	NF	0.5	0.0	CY	CY	GO:0005737	NP
947	WP_000453225.1	_	glycoside hydrolase family 3 protein	728.5	8.6	0.1	0.0	CY	CY	GO:0005886	NP
948	WP_001181162.1	_	Fe-S cluster assembly protein SufD	16.2	NF	0.2	0.0	CY	CY	GO:0005737	NP

949	WP_000277632.1	_	long-chain fatty acid--CoA ligase	561.8	3.6	3.0	0.0	IM	CY	GO:0005737	NP
950	WP_001000160.1	LIC_10159	tRNA uridine-5-carboxymethylaminomethyl(34) synthesis GTPase MnmE	9.4	NF	0.1	0.0	CY	CY	GO:0005737	NP
951	WP_000933331.1	LIC_10227	CHAT domain-containing protein	35.5	0.2	0.2	0.0	CY	OM	GO:0005737	P
952	WP_000824202.1	_	M23 family peptidase	12.5	0.1	NF	0.0	UN	CY	GO:0005615	NP
953	WP_001174417.1	LIC_12934	mannose-6-phosphate isomerase	54.7	0.6	NF	0.0	CY	CY	GO:0005737	NP
954	WP_001243368.1	LIC_10258	membrane protein	46.7	0.1	0.4	0.0	UN	OM	GO:0005615	P
955	WP_000827840.1	LIC_10879	hypothetical protein	1321.9	NF	14.7	0.0	UN	PP	GO:0005886	P
956	WP_000392647.1	LIC_11712	MULTISPECIES: magnesium transporter	65.2	NF	0.7	0.0	IM	IM	GO:0005737	NP
957	WP_001082180.1	LIC_10073	class I SAM-dependent methyltransferase	113.2	NF	1.2	0.0	CY	CY	GO:0005737	NP
958	WP_000284122.1	_	MULTISPECIES: acyl-CoA dehydrogenase	567.0	NF	6.2	0.0	CY	CY	GO:0005737	NP
959	WP_000410868.1	_	M23 family peptidase	27.6	0.3	NF	0.0	CY	CY	GO:0005615	NP
960	WP_000875950.1	_	DUF2505 domain-containing protein	3063.2	33.2	NF	0.0	CY	CY	GO:0005737	P
961	WP_000524416.1	LIC_12742	MULTISPECIES: NADH-quinone oxidoreductase subunit B	139.4	NF	1.5	0.0	IM	PP	GO:0005737	NP
962	WP_000428155.1	LIC_12575	TolC family protein	4075.4	4.3	39.7	0.0	OM	OM	GO:0005737	P
963	WP_000658458.1	LIC_12771	gamma-glutamyl-phosphate reductase	110.9	0.6	0.6	0.0	CY	CY	GO:0005737	NP
964	WP_000829707.1	LIC_11630	long-chain fatty acid--CoA ligase	587.0	2.0	4.2	0.0	CY	CY	GO:0005737	NP
965	WP_000490514.1	_	hybrid sensor histidine kinase/response regulator	24.8	NF	0.3	0.0	IM	OM	GO:0005737	NP

966	WP_000612198.1	_	3-phosphoserine/phosphohydroxythreonine transaminase	147.3	1.5	NF	0.0	CY	CY	GO:0005886	NP
967	WP_000535887.1	LIC_13451	flagellar hook-associated protein FlgK	63.6	0.6	NF	0.0	EC	EC	GO:0005737	P
968	WP_001042619.1	LIC_12845	50S ribosomal protein L17	1986.3	NF	19.8	0.0	CY	CY	GO:0005737	NP
969	WP_000157730.1	LIC_11389	flagellar protein FlbB	69.7	0.7	NF	0.0	UN	CY	GO:0005886	NP
970	WP_000200272.1	LIC_13275	hypothetical protein	64.3	0.6	NF	0.0	CY	CY	GO:0005737	NP
971	WP_001124828.1	LIC_13282	DNA ligase (NAD(+)) LigA	106.3	0.8	0.2	0.0	CY	CY	GO:0005737	NP
972	WP_000449386.1	LIC_11045	guanylate cyclase	34.8	NF	0.3	0.0	CY	CY	GO:0005737	NP
973	WP_000534971.1	LIC_13178	MULTISPECIES: acylphosphatase	173.2	1.7	NF	0.0	CY	CY	GO:0005737	NP
974	WP_000761157.1	LIC_11996	hypothetical protein	124.0	1.2	NF	0.0	UN	OM	GO:0005737	P
975	WP_000721859.1	_	3-deoxy-manno-octulosonate cytidylyltransferase	108.0	NF	1.0	0.0	CY	CY	GO:0005737	NP
976	WP_000986372.1	LIC_11188	flagellar hook protein FlgE	52.9	0.5	NF	0.0	EC	EC	GO:0005737	P
977	WP_000083101.1	LIC_10931	restriction endonuclease subunit S	26.0	0.2	NF	0.0	CY	OM	GO:0005737	NP
978	WP_000626203.1	LIC_13443	hypothetical protein	85.0	NF	0.8	0.0	UN	PP	GO:0005737	NP
979	WP_000598571.1	LIC_13316	ABC transporter ATP-binding protein	238.6	NF	2.3	0.0	CY	CY	GO:0005737	P
980	WP_000564919.1	LIC_11518	biotin carboxylase	232.4	2.2	NF	0.0	CY	CY	GO:0005737	NP
981	WP_000050477.1	LIC_11657	MULTISPECIES: flagellar export chaperone FliS	97.3	0.9	NF	0.0	CY	CY	GO:0005737	P
982	WP_001133822.1	_	acetyl-coenzyme A synthetase	243.0	1.2	1.0	0.0	CY	CY	GO:0005737	NP

983	WP_000861511.1	LIC_10557	response regulator	135.1	NF	1.2	0.0	CY	CY	GO:0005737	NP
984	WP_000834388.1	LIC_20264	pyruvate kinase	201.3	1.7	0.2	0.0	CY	CY	GO:0005737	NP
985	WP_001005981.1	LIC_11853	homoserine O-acetyltransferase	30.1	0.3	NF	0.0	CY	OM	GO:0005737	NP
986	WP_000410918.1	_	histone deacetylase	64.4	0.6	NF	0.0	CY	CY	GO:0005737	NP
987	WP_001012308.1	LIC_20243	hypothetical protein	9.0	0.1	NF	0.0	UN	CY	GO:0005737	P
988	WP_001229174.1	LIC_10459	adenosine deaminase	113.3	1.0	0.0	0.0	CY	CY	GO:0005737	NP
989	WP_000168949.1	LIC_11356	MULTISPECIES: ATP-dependent Clp protease adaptor ClpS	73.7	0.7	NF	0.0	CY	CY	GO:0005737	NP
990	WP_000063680.1	_	Fe-S-cluster-containing hydrogenase	1103.9	NF	9.7	0.0	IM	IM	GO:0005886	NP
991	WP_000004170.1	LIC_10523	molecular chaperone DnaJ	778.0	0.9	6.0	0.0	CY	CY	GO:0005737	NP
992	WP_002072371.1	LIC_12679	phosphomethylpyrimidine synthase	74.4	0.6	NF	0.0	CY	CY	GO:0005737	NP
993	WP_000798179.1	_	hypothetical protein	191.5	1.7	NF	0.0	UN	CY	GO:0005615	NP
994	WP_001973770.1	_	acetyl-CoA carboxylase carboxyltransferase subunit	626.7	4.3	1.1	0.0	CY	OM	GO:0005737	NP
995	WP_001979242.1	_	hypothetical protein	1147.6	4.5	5.3	0.0	UN	CY	GO:0019867	NP
996	WP_000612786.1	LIC_12455	chemotaxis response regulator protein-glutamate methylesterase	27.9	0.2	NF	0.0	CY	CY	GO:0005737	NP
997	WP_000921250.1	LIC_11777	biotin synthase	73.3	0.2	0.4	0.0	CY	CY	GO:0005737	NP
998	WP_000406879.1	LIC_11516	anti-sigma factor antagonist	194.6	1.6	NF	0.0	CY	CY	GO:0005737	NP
999	WP_000744009.1	LIC_10473	valine--tRNA ligase	238.6	1.6	0.4	0.0	CY	CY	GO:0005886	NP

1000	WP_000348685.1	LIC_10027	hypothetical protein	1547.5	NF	12.6	0.0	IM	IM	GO:0005886	P
1001	WP_000807170.1	_	hypothetical protein	236.1	NF	1.9	0.0	UN	CY	GO:0005737	NP
1002	WP_000427711.1	LIC_12855	MULTISPECIES: 50S ribosomal protein L30	341.0	NF	2.8	0.0	UN	CY	GO:0005737	NP
1003	WP_000345920.1	LIC_12001	hypothetical protein	699.5	NF	5.6	0.0	IM	IM	GO:0005886	NP
1004	WP_001075308.1	_	hypothetical protein	347.4	2.8	NF	0.0	CY	CY	GO:0005737	NP
1005	WP_001290040.1	_	WYL domain-containing transcriptional regulator	97.8	0.1	0.7	0.0	CY	CY	GO:0005737	NP
1006	WP_000626010.1	LIC_10484	threonine synthase	183.6	NF	1.4	0.0	UN	OM	GO:0005737	NP
1007	WP_000615236.1	LIC_11834	sigma factor regulatory protein FecR	229.3	1.8	NF	0.0	CY	CY	GO:0005615	NP
1008	WP_000660163.1	_	hypothetical protein	151.1	0.1	1.0	0.0	CY	OM	GO:0005615	NP
1009	WP_000102225.1	LIC_13348	DNA gyrase subunit B	159.7	1.2	NF	0.0	CY	CY	GO:0005737	NP
1010	WP_000377539.1	_	UDP-N-acetylmuramate--L-alanine ligase	12.8	0.1	NF	0.0	CY	CY	GO:0005886	NP
1011	WP_000097761.1	LIC_10231	hypothetical protein	175.4	1.3	NF	0.0	UN	OM	GO:0005886	P
1012	WP_000623186.1	LIC_11148	Holliday junction ATP-dependent DNA helicase RuvA	42.6	NF	0.3	0.0	CY	CY	GO:0005737	NP
1013	WP_000050531.1	LIC_20148	biliverdin-producing heme oxygenase	103.7	0.8	NF	0.0	UN	CY	GO:0005737	NP
1014	WP_001053743.1	LIC_13310	phenylalanine--tRNA ligase subunit alpha	184.1	0.9	0.5	0.0	CY	CY	GO:0005737	NP
1015	WP_001029784.1	LIC_20096	hypothetical protein	1240.4	NF	9.1	0.0	CY	OM	GO:0005737	P
1016	WP_000343398.1	LIC_12131	WxcM-like domain-containing protein	71.2	NF	0.5	0.0	UN	CY	GO:0005737	NP
1017	WP_000377275.1	LIC_11444	diguanylate cyclase response regulator	61.4	0.4	NF	0.0	CY	CY	GO:0005737	NP

1018	WP_000686200.1	LIC_10681	phosphoribosylformylglycinamidine synthase subunit PurQ	75.6	NF	0.5	0.0	CY	CY	GO:0005737	NP
1019	WP_000709350.1	_	flagellar type III secretion system protein FlhB	1.1	0.0	NF	0.0	IM	IM	GO:0005886	P
1020	WP_000414808.1	LIC_13183	MULTISPECIES: hypothetical protein	876.0	1.4	4.9	0.0	CY	CY	GO:0005737	NP
1021	WP_000205143.1	LIC_11313	peptidase M24 family protein	148.8	1.1	NF	0.0	CY	CY	GO:0005737	NP
1022	WP_000410299.1	_	MoxR family ATPase	81.9	NF	0.6	0.0	CY	CY	GO:0005737	P
1023	WP_000172726.1	LIC_13137	MULTISPECIES: sensor domain-containing diguanylate cyclase	67.8	0.5	NF	0.0	IM	CY	GO:0005886	NP
1024	WP_001173870.1	LIC_10483	membrane protein	326.8	1.4	0.9	0.0	UN	PP	GO:0005737	NP
1025	WP_000277765.1	LIC_10876	DUF3341 domain-containing protein	789.5	NF	5.6	0.0	UN	CY	GO:0005886	NP
1026	WP_000449876.1	LIC_11105	methylmalonyl-CoA mutase	113.4	NF	0.8	0.0	IM	OM	GO:0005737	NP
1027	WP_000836305.1	LIC_10766	lipoprotein	166.8	1.2	NF	0.0	UN	CY	GO:0005615	P
1028	WP_000895444.1	LIC_12422	amino acid aminotransferase	444.5	NF	3.1	0.0	CY	CY	GO:0005737	NP
1029	WP_000200035.1	LIC_10548	MULTISPECIES: uracil-DNA glycosylase	149.8	0.6	0.4	0.0	CY	CY	GO:0005737	NP
1030	WP_000076846.1	LIC_10006	DNA gyrase subunit A	189.2	1.1	0.2	0.0	CY	CY	GO:0005737	NP
1031	WP_000947684.1	LIC_11672	enoyl-CoA hydratase	293.9	2.0	NF	0.0	CY	CY	GO:0005737	NP
1032	WP_000547427.1	LIC_12161	6-phosphogluconolactonase	143.5	1.0	NF	0.0	CY	CY	GO:0005737	NP
1033	WP_000505733.1	LIC_12725	enoyl-CoA hydratase/isomerase family protein	348.7	1.9	0.4	0.0	CY	CY	GO:0005737	NP
1034	WP_000157338.1	_	hypothetical protein	136.7	NF	0.9	0.0	UN	CY	GO:0005737	NP

1035	WP_000135393.1	LIC_12396	amidohydrolase	89.6	0.1	0.5	0.0	UN	OM	GO:0005737	P
1036	WP_000179030.1	LIC_12941	excinuclease ABC subunit B	69.3	0.4	NF	0.0	CY	CY	GO:0005737	NP
1037	WP_000649925.1	LIC_10203	dTDP-glucose 4,6-dehydratase	23.8	NF	0.1	0.0	CY	CY	GO:0005737	NP
1038	WP_000610519.1	LIC_10011	lipoprotein LipL21	36758.6	91.6	132.8	0.0	PP	PP	GO:0005615	P
1039	WP_001188723.1	_	universal stress protein	134.8	NF	0.8	0.0	CY	CY	GO:0005737	NP
1040	WP_000626346.1	_	oxidoreductase	201.2	NF	1.2	0.0	CY	CY	GO:0005886	NP
1041	WP_000939462.1	LIC_13041	DoxX family protein	842.2	NF	5.1	0.0	IM	IM	GO:0005886	NP
1042	WP_000683676.1	LIC_12901	DUF4132 domain-containing protein	15.4	0.0	0.1	0.0	EC	OM	GO:0005886	P
1043	WP_000034360.1	LIC_10600	MaoC family dehydratase	796.6	2.8	2.0	0.0	CY	CY	GO:0005737	NP
1044	WP_000716642.1	_	hypothetical protein	3349.5	5.5	14.5	0.0	UN	CY	GO:0005737	P
1045	WP_001047667.1	LIC_13065	DUF2804 domain-containing protein	25.0	0.1	NF	0.0	UN	OM	GO:0005737	NP
1046	WP_000235141.1	LIC_11242	ATP synthase subunit gamma	827.7	NF	4.9	0.0	CY	CY	GO:0005737	NP
1047	WP_000492632.1	LIC_12101	cinA-like protein	55.4	0.3	NF	0.0	CY	OM	GO:0005737	NP
1048	WP_000805391.1	_	ferredoxin--NADP(+) reductase	532.4	3.1	NF	0.0	IM	CY	GO:0005737	NP
1049	WP_002068111.1	LIC_11452	exodeoxyribonuclease III	26.3	0.2	NF	0.0	CY	CY	GO:0005737	NP
1050	WP_000581182.1	LIC_10215	PIN/TRAM domain-containing protein	1709.1	NF	9.9	0.0	IM	IM	GO:0005886	NP
1051	WP_000672155.1	LIC_12231	hypothetical protein	251.7	NF	1.5	0.0	UN	IM	GO:0005615	P
1052	WP_000279894.1	LIC_11898	pyruvate dehydrogenase complex E1 component subunit beta	276.5	NF	1.6	0.0	CY	CY	GO:0005737	NP

1053	WP_000537600.1	LIC_10142	MULTISPECIES: NADH-quinone oxidoreductase subunit I	109.2	NF	0.6	0.0	CY	CY	GO:0005737	NP
1054	WP_000068280.1	LIC_11177	hypothetical protein	124.9	0.7	NF	0.0	UN	CY	GO:0005737	P
1055	WP_000050920.1	LIC_12002	succinate dehydrogenase flavoprotein subunit	982.7	NF	5.5	0.0	IM	PP	GO:0005737	NP
1056	WP_001272390.1	LIC_11844	paraslipin	188.4	NF	1.0	0.0	CY	CY	GO:0005886	NP
1057	WP_000447937.1	LIC_12371	hypothetical protein	138.5	0.8	NF	0.0	CY	CY	GO:0005737	NP
1058	WP_000099241.1	LIC_12786	indole-3-glycerol-phosphate synthase	42.1	0.2	NF	0.0	CY	CY	GO:0005737	NP
1059	WP_001228945.1	LIC_12966	lipoprotein LipL41	66302.1	59.2	284.8	0.0	UN	CY	GO:0005615	P
1060	WP_000661932.1	LIC_12154	SAM-dependent methyltransferase	33.7	NF	0.2	0.0	CY	CY	GO:0005737	NP
1061	WP_000538654.1	LIC_12508	histidinol-phosphate aminotransferase	68.1	NF	0.3	0.0	CY	CY	GO:0005737	NP
1062	WP_001016431.1	LIC_12676	WGR domain-containing protein	14.3	0.1	NF	0.0	CY	CY	GO:0005737	P
1063	WP_000868621.1	LIC_10452	glycosyltransferase family 2 protein	459.8	NF	2.3	0.0	IM	CY	GO:0005737	NP
1064	WP_000612432.1	LIC_12449	3-phosphoshikimate 1-carboxyvinyltransferase	29.3	0.1	NF	0.0	CY	CY	GO:0005737	NP
1065	WP_000364422.1	LIC_12003	succinate dehydrogenase/fumarate reductase iron-sulfur subunit	602.5	NF	2.9	0.0	CY	CY	GO:0005737	NP
1066	WP_001982425.1	_	alanine racemase	85.1	NF	0.4	0.0	CY	CY	GO:0005737	NP
1067	WP_001015901.1	_	phospho-sugar mutase	87.7	0.4	NF	0.0	CY	OM	GO:0005737	NP
1068	WP_000662000.1	LIC_12102	arginine--tRNA ligase	63.9	0.3	NF	0.0	CY	CY	GO:0005737	NP

1069	WP_001079033.1	LIC_11885	lipoprotein LipL46	21890.8	33.6	71.3	0.0	UN	OM	GO:0005615	P
1070	WP_000623631.1	LIC_11074	hypothetical protein	341.8	1.6	NF	0.0	UN	OM	GO:0005737	P
1071	WP_000691890.1	_	pirin family protein	107.9	0.5	NF	0.0	CY	CY	GO:0005737	NP
1072	WP_000620895.1	LIC_13407	ATPase	200.3	0.9	NF	0.0	CY	CY	GO:0005737	NP
1073	WP_000591983.1	LIC_10574	amidohydrolase	60.5	0.3	NF	0.0	CY	CY	GO:0005737	NP
1074	WP_000667978.1	LIC_11320	hypothetical protein	156.9	0.7	NF	0.0	UN	OM	GO:0005737	P
1075	WP_000390497.1	_	phosphoribosylanthranilate isomerase	33.5	NF	0.2	0.0	CY	CY	GO:0005737	NP
1076	WP_000243426.1	LIC_11239	ATP synthase subunit B	843.5	NF	3.9	0.0	UN	CY	GO:0005886	NP
1077	WP_000991320.1	LIC_11569	general secretion pathway protein GspC	87.2	NF	0.4	0.0	UN	OM	GO:0005886	P
1078	WP_000150993.1	LIC_11071	hypothetical protein	207.0	0.9	NF	0.0	UN	CY	GO:0005737	P
1079	WP_000390240.1	_	hydrolase	44.7	NF	0.2	0.0	CY	CY	GO:0005737	NP
1080	WP_000617710.1	LIC_12969	cell division protein FtsZ	40.1	NF	0.2	0.0	CY	EC	GO:0005737	NP
1081	WP_001054085.1	LIC_11573	type II secretion system protein GspG	624.9	2.8	NF	0.0	IM	PP	GO:0005886	P
1082	WP_001095358.1	LIC_10546	alpha/beta hydrolase	361.8	NF	1.6	0.0	UN	OM	GO:0005615	NP
1083	WP_001119768.1	LIC_13026	DJ-1/PfpI family protein	198.0	NF	0.8	0.0	UN	CY	GO:0005737	NP
1084	WP_001142685.1	_	aldehyde dehydrogenase	390.3	NF	1.7	0.0	CY	CY	GO:0005737	NP
1085	WP_000603361.1	LIC_12311	MULTISPECIES: ABC transporter ATP-binding protein	28.5	NF	0.1	0.0	IM	CY	GO:0005737	P
1086	WP_000819152.1	_	hypothetical protein	9328.4	25.1	13.1	0.0	UN	EC	GO:0005737	P

1087	WP_000559149.1	LIC_11993	MULTISPECIES: hypothetical protein	110.2	0.4	NF	0.0	CY	CY	GO:0005737	NP
1088	WP_000863354.1	_	cAMP-binding protein	84.0	0.1	0.3	0.0	UN	OM	GO:0005737	NP
1089	WP_000570129.1	LIC_12028	cysteine--tRNA ligase	63.1	0.2	NF	0.0	CY	CY	GO:0005737	NP
1090	WP_000052101.1	LIC_10936	type I-B CRISPR-associated protein Cas7/Cst2/DevR	112.7	0.4	NF	0.0	CY	CY	GO:0005737	NP
1091	WP_001221811.1	LIC_10357	tryptophan--tRNA ligase	96.9	0.4	NF	0.0	CY	PP	GO:0005737	NP
1092	WP_000514163.1	LIC_10859	MULTISPECIES: tryptophan synthase subunit beta	207.2	NF	0.8	0.0	CY	CY	GO:0005737	NP
1093	WP_000487322.1	LIC_12151	CDP-glucose 4,6-dehydratase	168.1	NF	0.6	0.0	CY	CY	GO:0005886	NP
1094	WP_001072290.1	LIC_12544	DUF4115 domain-containing protein	1295.1	1.0	3.6	0.0	UN	OM	GO:0005737	NP
1095	WP_000234594.1	_	hypothetical protein	97.4	NF	0.3	0.0	UN	CY	GO:0005737	NP
1096	WP_000204770.1	LIC_13371	sulfate adenylyltransferase subunit CysD	143.3	0.5	NF	0.0	CY	CY	GO:0005737	NP
1097	WP_000802798.1	LIC_11189	HD family phosphohydrolase	63.5	0.2	NF	0.0	CY	CY	GO:0005737	NP
1098	WP_000045629.1	LIC_10662	polyketide cyclase	943.9	1.6	1.6	0.0	IM	PP	GO:0005886	NP
1099	WP_001270455.1	LIC_12347	50S ribosomal protein L11 methyltransferase	42.2	NF	0.1	0.0	CY	CY	GO:0005886	NP
1100	WP_000529175.1	LIC_13354	hypothetical protein	1294.9	4.3	NF	0.0	UN	EC	GO:0005737	P
1101	WP_001222086.1	LIC_11752	glycosyltransferase family 4 protein	121.3	NF	0.4	0.0	CY	CY	GO:0005737	NP
1102	WP_000018115.1	LIC_11954	enolase	287.5	NF	0.9	0.0	CY	CY	GO:0005737	NP
1103	WP_000687983.1	LIC_10380	GyrI-like domain-containing protein	15468.3	19.8	29.4	0.0	IM	CY	GO:0005886	NP

1104	WP_000908508.1	_	MULTISPECIES: anti-sigma factor antagonist	48.0	NF	0.2	0.0	UN	CY	GO:0005737	NP
1105	WP_000017090.1	LIC_11570	type II secretion system protein GspD	296.3	0.9	NF	0.0	OM	OM	GO:0019867	P
1106	WP_000281386.1	_	hypothetical protein	506.2	0.9	0.6	0.0	UN	OM	GO:0005886	NP
1107	WP_000677438.1	LIC_10125	tetratricopeptide repeat protein	1208.6	1.4	2.1	0.0	UN	OM	GO:0005615	P
1108	WP_000027575.1	LIC_12500	methyl-accepting chemotaxis protein	81.9	NF	0.2	0.0	UN	OM	GO:0005886	P
1109	WP_001289449.1	LIC_11998	alpha/beta hydrolase	152.7	0.4	NF	0.0	CY	CY	GO:0005737	NP
1110	WP_000979308.1	LIC_11490	hypothetical protein	111.1	NF	0.3	0.0	CY	CY	GO:0005886	NP
1111	WP_001053085.1	LIC_13257	alcohol dehydrogenase	585.4	NF	1.6	0.0	CY	CY	GO:0005737	NP
1112	WP_001038998.1	LIC_10432	MULTISPECIES: ATP-dependent metallopeptidase FtsH/Yme1/Tma family protein	428.9	0.2	1.0	0.0	IM	IM	GO:0005886	NP
1113	WP_001177028.1	LIC_13181	SET domain-containing protein-lysine N-methyltransferase	94.1	NF	0.3	0.0	CY	CY	GO:0005737	P
1114	WP_000372281.1	LIC_12516	acetoacetate--CoA ligase	64.8	0.2	NF	0.0	CY	OM	GO:0005737	P
1115	WP_001165152.1	LIC_11699	glycerol-3-phosphate dehydrogenase/oxidase	1002.6	NF	2.6	0.0	CY	CY	GO:0005737	NP
1116	WP_000062490.1	LIC_11546	HPr kinase/phosphorylase	64.4	NF	0.2	0.0	CY	CY	GO:0005737	NP
1117	WP_000683294.1	LIC_11318	amidohydrolase	134.1	0.3	NF	0.0	CY	CY	GO:0005737	NP
1118	WP_000810942.1	_	hypothetical protein	938.2	0.3	2.1	0.0	CY	CY	GO:0005737	NP
1119	WP_000390247.1	_	hypothetical protein	20.7	NF	0.1	0.0	CY	CY	GO:0005737	NP
1120	WP_000045934.1	_	hypothetical protein	9.3	0.0	NF	0.0	CY	OM	GO:0005737	NP

1121	WP_000102005.1	LIC_10209	cytochrome c oxidase subunit I	1637.1	NF	3.9	0.0	IM	IM	GO:0005886	NP
1122	WP_000483735.1	LIC_10985	hypothetical protein	489.7	1.2	NF	0.0	CY	OM	GO:0005886	P
1123	WP_001243475.1	LIC_12708	hypothetical protein	158.4	NF	0.4	0.0	UN	OM	GO:0005886	P
1124	WP_001971487.1	_	TlpA family protein disulfide reductase	193.1	NF	0.5	0.0	UN	OM	GO:0005615	P
1125	WP_000757100.1	_	MULTISPECIES: TIGR04454 family lipoprotein	19397.7	17.7	27.0	0.0	UN	PP	GO:0005615	NP
1126	WP_000931842.1	_	preprotein translocase subunit SecG	1014.9	NF	2.3	0.0	IM	PP	GO:0005886	NP
1127	WP_000234667.1	LIC_11831	GMP synthetase	85.8	0.2	NF	0.0	CY	CY	GO:0005737	NP
1128	WP_000768212.1	LIC_20229	phosphate ABC transporter	653.0	NF	1.4	0.0	IM	CY	GO:0005615	NP
1129	WP_001074690.1	_	short-chain dehydrogenase	952.3	NF	2.1	0.0	IM	IM	GO:0005886	NP
1130	WP_000251310.1	LIC_11028	biopolymer transporter TolR	51.4	NF	0.1	0.0	UN	OM	GO:0005615	P
1131	WP_001089694.1	LIC_12285	potassium transporter	784.6	NF	1.7	0.0	IM	IM	GO:0005886	P
1132	WP_000697460.1	LIC_11606	phosphohydrolase	91.9	NF	0.2	0.0	CY	CY	GO:0005737	NP
1133	WP_000721547.1	LIC_12353	hypothetical protein	587.7	1.2	NF	0.0	CY	CY	GO:0005615	NP
1134	WP_000698133.1	_	alpha/beta hydrolase	237.9	NF	0.5	0.0	UN	OM	GO:0005886	P
1135	WP_000291493.1	LIC_12693	TolC family protein	2038.3	0.5	3.8	0.0	UN	OM	GO:0005615	P
1136	WP_000878646.1	_	cytochrome c	5386.0	NF	11.0	0.0	UN	PP	GO:0005737	P
1137	WP_000501992.1	LIC_10624	endoflagellar basal body-associated protein	1476.8	2.8	0.2	0.0	UN	CY	GO:0005886	P
1138	WP_000582618.1	LIC_12169	acetylneuraminic acid synthetase	65.7	NF	0.1	0.0	CY	CY	GO:0005737	P

1139	WP_000058904.1	LIC_12268	dehydrogenase	41.9	0.1	0.0	0.0	CY	CY	GO:0005737	NP
1140	WP_000643183.1	_	carbamoyl-phosphate synthase small subunit	246.3	NF	0.5	0.0	CY	CY	GO:0005737	NP
1141	WP_000377029.1	LIC_10226	hypothetical protein	173.3	NF	0.3	0.0	CY	OM	GO:0005737	P
1142	WP_001292110.1	LIC_20098	XerD	190.1	NF	0.3	0.0	CY	OM	GO:0005737	NP
1143	WP_000651714.1	LIC_11782	oxidoreductase	2459.1	1.2	3.2	0.0	CY	CY	GO:0005886	NP
1144	WP_001071110.1	LIC_11553	PASTA domain-containing protein	654.7	NF	1.1	0.0	CY	OM	GO:0005737	NP
1145	WP_000723090.1	LIC_12124	dTDP-glucose 4,6-dehydratase	112.7	NF	0.2	0.0	CY	CY	GO:0005737	NP
1146	WP_000431423.1	LIC_11106	1-acyl-sn-glycerol-3-phosphate acyltransferase	53.9	NF	0.1	0.0	CY	CY	GO:0005737	NP
1147	WP_001082055.1	LIC_10325	tetratricopeptide repeat protein	288.0	NF	0.5	0.0	UN	OM	GO:0005615	P
1148	WP_000114512.1	LIC_12570	tRNA (N6-isopentenyl adenosine(37)-C2)-methylthiotransferase MiaB	58.4	NF	0.1	0.0	CY	CY	GO:0005737	NP
1149	WP_001288293.1	_	MULTISPECIES: hypothetical protein	194.0	0.3	NF	0.0	UN	CY	GO:0005737	P
1150	WP_001181792.1	_	biotin--[acetyl-CoA-carboxylase] ligase	199.9	0.3	NF	0.0	CY	CY	GO:0005886	NP
1151	WP_000448656.1	LIC_20010	porphobilinogen synthase	129.6	0.2	NF	0.0	CY	OM	GO:0005737	NP
1152	WP_000711420.1	_	MULTISPECIES: lipoprotein, tandem type	4502.9	0.4	5.6	0.0	CY	CY	GO:0005737	NP
1153	WP_000793300.1	LIC_13360	DUF4139 domain-containing protein	67.7	NF	0.1	0.0	UN	OM	GO:0005737	P
1154	WP_000680449.1	LIC_12834	hydrolase	502.2	0.6	NF	0.0	CY	CY	GO:0005737	P
1155	WP_000846596.1	_	hypothetical protein	340.6	0.4	NF	0.0	UN	OM	GO:0005615	P
1156	WP_000689396.1	_	hypothetical protein	1281.6	0.4	1.1	0.0	CY	OM	GO:0005737	P

1157	WP_001013490.1	_	peptidase	1116.4	0.6	0.7	0.0	CY	CY	GO:0005615	NP
1158	WP_000459252.1	_	protein translocase subunit SecF	165.9	NF	0.2	0.0	IM	IM	GO:0005886	NP
1159	WP_001166217.1	LIC_12827	hypothetical protein	59.3	NF	0.1	0.0	CY	CY	GO:0005737	NP
1160	WP_000645315.1	LIC_12388	cyclic nucleotide-binding domain-containing protein	184.3	NF	0.2	0.0	CY	CY	GO:0005737	NP
1161	WP_000504735.1	LIC_20028	NAD(P)/FAD-dependent oxidoreductase	458.9	NF	0.4	0.0	IM	CY	GO:0005737	NP
1162	WP_000688483.1	LIC_11549	sigma-54-dependent Fis family transcriptional regulator	50.1	0.0	NF	0.0	CY	CY	GO:0005737	P
1163	WP_000390190.1	LIC_10157	protein translocase component YidC	929.9	NF	0.7	0.0	IM	OM	GO:0005886	NP
1164	WP_002077265.1	LIC_11500	type I methionyl aminopeptidase	120.5	0.1	NF	0.0	CY	CY	GO:0005737	NP
1165	WP_000398444.1	LIC_10608	Lon protease	95.3	NF	0.1	0.0	CY	CY	GO:0005737	NP
1166	WP_000045337.1	_	hypothetical protein	126.3	0.1	NF	0.0	CY	CY	GO:0005737	NP
1167	WP_001211481.1	_	hypothetical protein	945.9	0.7	NF	0.0	UN	CY	GO:0005615	NP
1168	WP_000809534.1	LIC_10009	lipoprotein LenA	1052.7	NF	0.8	0.0	UN	OM	GO:0005886	P
1169	WP_000616634.1	LIC_10134	DUF3095 domain-containing protein	305.4	NF	0.2	0.0	CY	CY	GO:0005737	NP
1170	WP_000447378.1	LIC_11845	paraslipin	413.8	0.3	NF	0.0	CY	CY	GO:0005886	NP
1171	WP_000356651.1	LIC_10405	tetratricopeptide repeat protein	304.1	0.2	NF	0.0	OM	OM	GO:0005886	P
1172	WP_000853744.1	LIC_11571	type II secretion system protein GspE	81.4	NF	0.1	0.0	CY	CY	GO:0005737	P
1173	WP_001086272.1	LIC_10628	cytochrome c	8734.5	2.0	2.9	0.0	UN	PP	GO:0005886	P

1174	WP_000783496.1	LIC_11587	vitamin B12-dependent ribonucleotide reductase	131.5	0.1	NF	0.0	CY	CY	GO:0005737	NP
1175	WP_000835640.1	LIC_12477	carboxypeptidase	407.9	NF	0.2	0.0	IM	OM	GO:0005886	NP
1176	WP_002145501.1	LIC_12691	hypothetical protein	4799.0	NF	2.2	0.0	UN	CY	GO:0005886	NP
1177	WP_000166979.1	LIC_11851	hypothetical protein	310.9	0.1	NF	0.0	OM	OM	GO:0005615	NP
1178	WP_000682856.1	LIC_11793	signal peptide peptidase SppA	1895.7	0.8	NF	0.0	IM	OM	GO:0005615	NP
1179	WP_001018338.1	_	(Fe-S)-binding protein	790.8	0.1	0.2	0.0	IM	IM	GO:0005886	NP
1180	WP_001064927.1	LIC_10873	MULTISPECIES: class III cytochrome C domain protein	392.6	0.1	NF	0.0	UN	PP	GO:0005886	NP
1181	WP_001231967.1	LIC_12576	hypothetical protein	349.8	0.1	NF	0.0	CY	OM	GO:0005886	P
1182	WP_000698820.1	LIC_12163	carbamoyltransferase	279.7	NF	0.1	0.0	CY	CY	GO:0005737	NP
1183	WP_001048225.1	_	OMA87-like protein	3655.0	1.0	0.1	0.0	UN	OM	GO:0005886	NP
1184	WP_000127254.1	LIC_10290	MULTISPECIES: NAD(P) transhydrogenase subunit beta	801.6	NF	0.2	0.0	IM	IM	GO:0005886	NP
1185	WP_001071434.1	LIC_10115	protein disulfide-isomerase	3738.8	NF	1.1	0.0	IM	OM	GO:0005886	NP
1186	WP_000670773.1	_	polynucleotide adenylyltransferase PcnB	309.9	NF	0.1	0.0	CY	CY	GO:0005737	NP
1187	WP_000027600.1	LIC_12921	methyl-accepting chemotaxis protein	295.7	0.1	NF	0.0	IM	OM	GO:0005886	P
1188	WP_001291001.1	LIC_10862	adenylate/guanylate cyclase domain-containing protein	838.5	NF	0.2	0.0	IM	IM	GO:0005886	NP
1189	WP_001145096.1	_	cytochrome c oxidase subunit II	1124.7	NF	0.3	0.0	IM	IM	GO:0005886	NP
1190	WP_001047263.1	LIC_11740	TldD/PmbA family protein	119.3	0.0	NF	0.0	CY	OM	GO:0005737	NP

1191	WP_001018374.1	LIC_11928	FAD-binding oxidoreductase	479.7	0.1	NF	0.0	UN	CY	GO:0005737	NP
1192	WP_000277382.1	LIC_20092	peptidase	42.6	0.0	NF	0.0	CY	OM	GO:0005737	NP
1193	WP_000459126.1	LIC_11820	cysteine synthase family protein	527.1	NF	0.1	0.0	CY	OM	GO:0005737	NP
1194	WP_000121860.1	_	sensor domain-containing diguanylate cyclase	49.5	0.0	NF	0.0	CY	CY	GO:0005737	P
1195	WP_000539242.1	LIC_11122	sigma factor regulatory protein FecR	1000.1	0.0	NF	0.0	UN	CY	GO:0005886	NP

Extracellular proteome analysis shows the abundance of Histidine kinase sensor protein (LIC_11528), DNA helicase (LIC_11624), Putative lipoprotein (LIC_10713) and Peptidase C39 domain protein (LIC_10511) in Leptospira interrogans grown in EMJH medium.

(Abhijit. Sarma, Dhandapani. Gunasekaran, D A B. Rex, Thoduvayil. Sikha, K Mangalaparthi. Kiran, M Pinto. Sneha, Prasad T S. Keshava, Madanan. Madathiparambil)

Correspondence: Email: madanan.mg@icmr.gov.in

Supplementary Table S2

KEGG based search for function of extracellular proteins:

Level-1	Level-2	Level-3	KEGG No	Cross-reference (KEGG)	Protein
Metabolism	Carbohydrate metabolism	Amino sugar and nucleotide sugar metabolism [PATH:lic00520]	K00820 glmS; glucosamine---fructose-6-phosphate aminotransferase (isomerizing) [EC:2.6.1.16]	lic:LIC_10445	LIC_10445
Metabolism	Energy metabolism	Oxidative phosphorylation [PATH:lic00190]	K02113 ATPF1D; F-type H+-transporting ATPase subunit delta	lic:LIC_11240	LIC_11240
Metabolism	Amino acid metabolism	Alanine, aspartate and glutamate metabolism [PATH:lic00250]	K00820 glmS; glucosamine---fructose-6-phosphate aminotransferase (isomerizing) [EC:2.6.1.16]	lic:LIC_10445	LIC_10445
Metabolism	Metabolism of cofactors and vitamins	Folate biosynthesis [PATH:lic00790]	K00950 folK; 2-amino-4-hydroxy-6-hydroxymethyldihydropteridine diphosphokinase [EC:2.7.6.3]	lic:LIC_13164	LIC_13164
Genetic Information Processing	Translation	Ribosome [PATH:lic03010]	K02959 RP-S16; small subunit ribosomal protein S16	lic:LIC_11555	LIC_11555
Genetic Information Processing	Folding, sorting and degradation	Protein export [PATH:lic03060]	K03210 yajC; preprotein translocase subunit YajC	lic:LIC_12540	LIC_12540

Genetic Information Processing	Replication and repair	Nucleotide excision repair [PATH:lic03420]	K03657 uvrD; DNA helicase II / ATP-dependent DNA helicase PcrA [EC:3.6.4.12]	lic:LIC_11624	LIC_11624
Genetic Information Processing	Replication and repair	Mismatch repair [PATH:lic03430]	K03657 uvrD; DNA helicase II / ATP-dependent DNA helicase PcrA [EC:3.6.4.12]	lic:LIC_11624	LIC_11624
Environmental Information Processing	Membrane transport	Bacterial secretion system [PATH:lic03070]	K03210 yajC; preprotein translocase subunit YajC	lic:LIC_12540	LIC_12540
Cellular Processes	Cellular community - prokaryotes	Quorum sensing [PATH:lic02024]	K03210 yajC; preprotein translocase subunit YajC	lic:LIC_12540	LIC_12540
Brite Hierarchies	Protein families: metabolism	Protein kinases [BR:lic01001]	K02484 K02484; two-component system, OmpR family, sensor kinase [EC:2.7.13.3]	lic:LIC_11528	LIC_11528
Brite Hierarchies	Protein families: metabolism	Peptidases [BR:lic01002]	K00820 glmS; glucosamine---fructose-6-phosphate aminotransferase (isomerizing) [EC:2.6.1.16]	lic:LIC_10445	LIC_10445
Brite Hierarchies	Protein families: metabolism	Photosynthesis proteins [BR:lic00194]	K02113 ATPF1D; F-type H+-transporting ATPase subunit delta	lic:LIC_11240	LIC_11240
Brite Hierarchies	Protein families: genetic information processing	Ribosome [BR:lic03011]	K02959 RP-S16; small subunit ribosomal protein S16	lic:LIC_11555	LIC_11555
Brite Hierarchies	Protein families: genetic information processing	DNA repair and recombination proteins [BR:lic03400]	K03657 uvrD; DNA helicase II / ATP-dependent DNA helicase PcrA [EC:3.6.4.12]	lic:LIC_11624	LIC_11624
Brite Hierarchies	Protein families: genetic information processing	Mitochondrial biogenesis [BR:lic03029]	K02959 RP-S16; small subunit ribosomal protein S16	lic:LIC_11555	LIC_11555
Brite Hierarchies	Protein families: signaling and cellular processes	Secretion system [BR:lic02044]	K03210 yajC; preprotein translocase subunit YajC	lic:LIC_12540	LIC_12540
Brite Hierarchies	Protein families: signaling and cellular processes	Two-component system [BR:lic02022]	K02484 K02484; two-component system, OmpR family, sensor kinase [EC:2.7.13.3]	lic:LIC_11528	LIC_11528

Not Included in Pathway or Brite	Unclassified: signaling and cellular processes	Others	K07231 K07231; putative iron-regulated protein	lic:LIC_10713	LIC_10713
